# Longitudinal association between neighborhood-level social capital and incidence of major psychiatric disorders in a cohort of 1.4 million people in Sweden

**DOI:** 10.1038/s44220-025-00518-z

**Published:** 2025-10-20

**Authors:** Angela Song-Chase, Jennifer Dykxhoorn, Anna-Clara Hollander, Cecilia Magnusson, Christina Dalman, James B. Kirkbride

**Affiliations:** 1 PsyLife Group, Division of Psychiatry, UCL, London, UK; 2 Department of Primary Care and Population Health, UCL, London, UK; 3 Department of Global Public Health, Karolinska Institutet, Stockholm, Sweden; 4 Centre for Epidemiology and Community Medicine, Stockholm County Council, Stockholm, Sweden

**Keywords:** Society, Risk factors

## Abstract

Social capital—the trust and cohesion within communities—has been linked to mental health, yet longitudinal evidence remains scarce. Here we show that neighborhood-level personal trust predicts the incidence of severe mental illness in a large, population-based cohort in Stockholm County, Sweden. Among 1.47 million Swedish-born residents followed over 15 years, higher personal trust at baseline was associated with reduced rates of non-affective psychotic disorder and bipolar disorder without psychosis over the follow-up period, but only among individuals of Swedish or European heritage. In contrast, the same exposure increased incidence rates among those of North African, Middle Eastern or Sub-Saharan African heritage. Political and welfare trust showed no consistent associations. These findings suggest that social capital may confer mental health benefits or risks depending on one’s own social position, highlighting the need for nuanced public mental health strategies that consider structural and cultural contexts in promoting mental wellbeing.

## Abstract (web summary)

The authors present data demonstrating that neighborhood-level personal trust influences severe mental illness incidence in a large cohort in Sweden, varying by heritage, emphasizing tailored public health strategies for mental wellbeing.

## Main

In many high-income countries, the incidence of non-affective psychotic disorders such as schizophrenia shows strong social gradients according to individual-level socioeconomic status^1^, school-level social fragmentation (that is, lack of cohesiveness)^2^ and neighborhood-level deprivation^3–5^, population density^5–7^, inequality^8^, residential instability^9^ and social fragmentation^10^. These patterns appear weaker for affective psychotic disorders such as bipolar disorder or depression with psychotic features^8,11,12^, as well as for bipolar disorder without psychosis^11^. Corollary patterns have been found with respect to ethnicity and migrant status^13^, where recent evidence suggests that excess psychosis risk for some ethnic minority and migrant groups is attributable to social gradients in health, including experiences of structural disadvantage^14,15^ and psychosocial disempowerment^14^. These findings lend credence to the possibility that access to social capital may be protective against psychosis.

Social capital encapsulates the shared resources, values and connections that enable a network of stakeholders to realize common goals or objectives^16^. Social capital has been variously conceptualized, but is regarded as a multidimensional construct that may be the property of individuals, groups or both (Extended Data Table 1). It is theorized to protect against mental health problems in two non-mutually exclusive ways^17^: (1) by providing strong social ties that promote and maintain healthy affective, cognitive and emotional states or (2) during periods of adversity, by acting as a buffer against stressors that may otherwise have deleterious effects on health.

Whether low social capital is a specific risk factor for psychosis remains unclear. So far, most studies have been cross-sectional and have adopted varying definitions of, or proxies for, social capital^18^. For example, studies in Ireland^3^ and Australia^19^ have suggested that cross-sectional associations exist between a higher proportion of neighborhood-level volunteerism and lower psychosis incidence, a finding restricted to non-affective psychotic disorders in one of these studies^19^. Two further cross-sectional studies, using neighborhood-level voter turnout at elections, have also reported similar associations with psychosis incidence^20,21^. By contrast, one cross-sectional study in the Netherlands found no association between neighborhood-level relational social capital (as rated by control participants) (Extended Data Table 1) and schizophrenia incidence^22^. A further study of relational social capital—as reported in a random, but over-representative sample of White participants in southeast London—found a nonlinear relationship with non-affective psychotic disorders incidence^23^, being higher in areas with either low or high (compared with moderate) levels of relational social capital. The authors theorized that higher levels of relational social capital as measured by a disproportionate number of White respondents might potentially be unavailable, exclusionary and even harmful for other ethnic groups. Further analysis supported this possibility, with a higher incidence in neighborhoods with higher relational social capital being even more pronounced in ethnic minority groups than the White group^23^. This suggests that relational social capital may only be protective when accessible, consistent with evidence of a protective effect of ethnic or migrant density from several studies, which have observed lower rates of psychotic disorders in ethnic minority or migrant groups who live in neighborhoods with a higher proportion of people with similar ethnic or migrant identities^8,24,25^.

So far, only one longitudinal study has investigated the longitudinal relationship between social capital and the incidence of psychotic disorders^26^, but follow-up was short (two years), voter turnout was a proxy for social capital, and the study was limited to inpatient hospitalization (for either psychotic disorder or depression). After multivariable adjustment, higher voter turnout was associated with a reduced hospitalization risk for psychosis, although null findings were reported for depression. Despite this, broader evidence^27^ (albeit predominantly cross-sectional) has suggested that greater social capital is associated with lower risks of other non-psychotic psychiatric problems. Limited longitudinal evidence also supports this possibility. For example, greater individual-level cognitive social capital has been associated with a lower risk of common mental disorders^27^, as well as fewer symptoms^28^. Further recent longitudinal work from Canada^29^ has also found that increased risks for four out of five adolescent mental health and behavioral symptoms associated with adverse childhood events were completely ameliorated among children growing up in socially cohesive neighborhoods, supporting a buffering role for social capital. In longitudinal research in Sweden, relational social capital also appears to mediate subsequent levels of psychological distress in refugees (detailed social capital measures available in the Stockholm Public Health Cohort (SPHC) survey^30^ were used). We are unaware of any study that has investigated the relationship between bipolar disorder and social capital as yet. In general, bipolar disorders—which may or may not present with psychotic features—tend to show less association, cross-sectionally^12^ or longitudinally^13^, with neighborhood social environments^5^.

To address the paucity of high-quality longitudinal research examining the role of social capital on major psychiatric disorders in a single study, we investigated whether time-varying exposures to various domains of social capital were prospectively associated with subsequent incidence of non-affective psychotic disorders, affective psychotic disorders and bipolar disorder without psychosis, as recorded in the Swedish national patient register. Population data were drawn from Psychiatry Sweden, a linked database of Swedish population registers following a cohort of over 1.4 million people followed for up to 15 years while living in Stockholm County, and linked to neighborhood social capital, independently derived from the SPHC survey in 2002^31^. We hypothesized that neighborhood-level relational social capital (operationalized as neighborhood levels of personal trust) would be (1) most strongly associated with reduced non-affective psychotic disorders incidence, compared with other psychiatric outcomes and other forms of social capital; (2) associated with reduced incidence in the majority Swedish-born population, but not necessarily for those with an immigrant background, if respondents to the SPHC survey were disproportionately of Swedish-born origin, consistent with related theoretical^32^ and empirical^23^ evidence.

## Results

### Sample characteristics and missing data

**Fig. 1:**
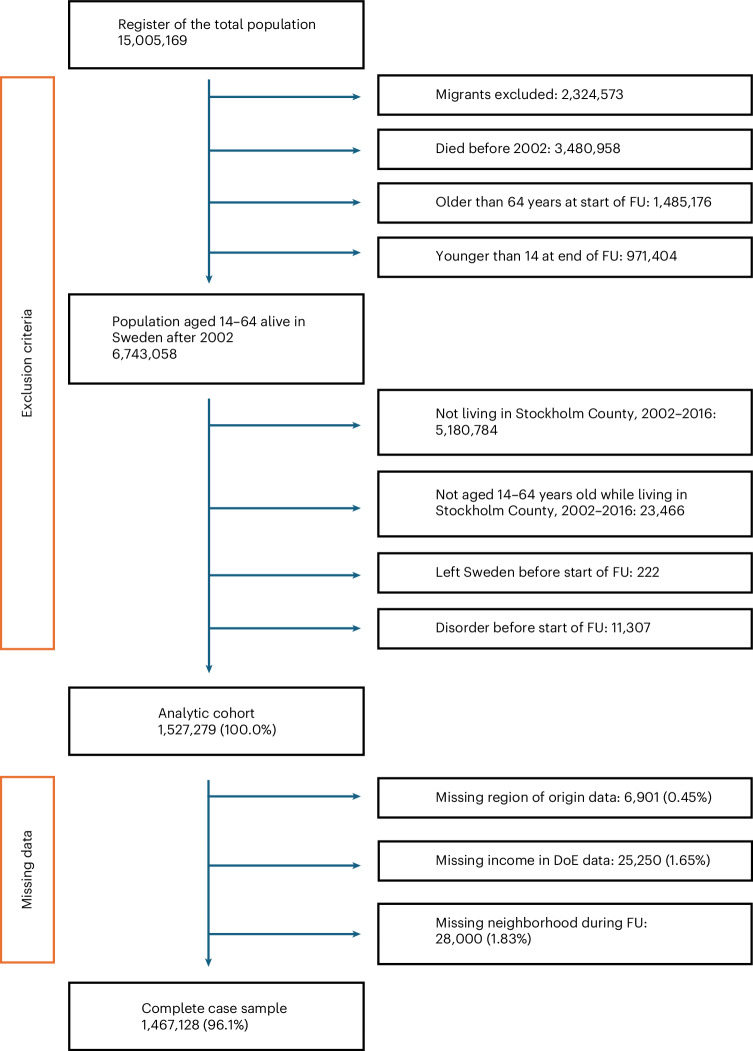
Flow chart of sample derivation. Flow chart of participants into the analytical cohort in these analyses. FU, follow-up; DoE, date of cohort entry.

The initial cohort included 1,527,279 participants aged between 14 and 64 years, with no previous diagnosis of any outcome of interest, and living in one of 890 small area marketing statistics (SAMS) neighborhoods in Stockholm County between 2002 and 2016. From this, we excluded 3.93% of participants (*N* = 60,151; Fig. 1) with missing data on parental region of origin (0.45%), income at cohort entry (1.65%) or neighborhood data during follow-up (1.83%), including participants living in one of 77 SAMS where no social capital data were available. Participants who were men, younger, children of migrants, without a personal or parental history of an outcome of interest, from lower income groups and from less deprived, more densely populated neighborhoods at cohort entry, and with lower median levels of trust were more likely to have missing data (all *P* ≤ 0.01; Supplementary Table 1).

The complete case cohort included 1,467,128 participants (96.07%) who were followed for 14,581,475 person-years, during which time we identified 17,760 cases with a first diagnosis of our primary or secondary outcomes in 813 neighborhoods in Stockholm County. This included 7,291 incidence cases of non-affective psychotic disorders (41.05%), 2,357 of affective psychotic disorders (13.27%) and 8,112 of bipolar disorder without psychosis (45.68%). In total, 50.14% of the cohort were male and 49.86% female (Table 1). Participants with non-affective psychotic disorders were more likely to be men, younger, children of migrants, non-Swedish origin (except Asian), have a parental history of an outcome of interest, belong to a lower family disposable income quintile, and live in the most densely populated and deprived SAMS quintiles at cohort entry (Table 1). Similar patterns were observed for secondary outcomes (Supplementary Tables 2 and 3) separately, except these outcomes were more common in women, and exhibited weaker gradients by population density at cohort entry.

**Table 1:** Complete case sample characteristics by primary outcome status

Variable	Population at risk^a^		Non-affective psychosis		Association	
	*N*	%	*N*	%	*χ*² (d.f.)^c^	*P*
Total	1,459,837	99.50	7,291	0.50		
Sex					109.8 (1)	<0.001
Male	731,935	99.44	4,104	0.56		
Female	727,902	99.56	3,187	0.44		
Age group (cohort exit)					1,076.2 (9)	<0.001
14–19	130,379	99.55	584	0.45		
20–24	169,314	99.36	1,097	0.64		
25–29	156,473	99.39	960	0.61		
30–34	127,869	99.38	796	0.62		
35–39	115,660	99.37	734	0.63		
40–44	118,253	99.37	754	0.63		
45–49	115,152	99.38	715	0.62		
50–54	116,178	99.46	629	0.54		
55–59	98,582	99.43	567	0.57		
60–64	311,977	99.85	455	0.15		
Migrant status					444.7 (1)	<0.001
Swedish-born	1,176,612	99.56	5,162	0.44		
Children of migrants	283,225	99.25	2,129	0.75		
Region-of-origin					491.9 (6)	<0.001
Sweden	1,176,612	99.56	5,162	0.44		
Other Europe	123,597	99.19	1,009	0.81		
Asia	5,488	99.65	19	0.35		
North Africa and Middle East	33,199	99.40	200	0.60		
Sub-Saharan Africa	8,203	99.11	74	0.89		
Mixed	106,638	99.27	784	0.73		
Other	6,100	99.30	43	0.70		
Parental history of SMI					947.3 (1)	<0.001
No	1,401,019	99.54	6,477	0.46		
Yes	58,818	98.63	814	1.37		
Family disposable income (cohort entry)^b^					2,335.3 (4)	<0.001
1 – Lowest	228,628	98.92	2,497	1.08		
2	245,299	99.40	1,490	0.60		
3	254,407	99.55	1,154	0.45		
4	245,328	99.62	942	0.38		
5 – Highest	486,175	99.75	1,208	0.25		
Population density (cohort entry)^b^					309.8 (4)	<0.001
1 – Lowest	9,249	99.66	32	0.34		
2	64,261	99.64	229	0.36		
3	91,626	99.64	327	0.36		
4	423,542	99.62	1,615	0.38		
5 – Highest	871,159	99.42	5,088	0.58		
Deprivation (cohort entry)^b^					919.8 (4)	<0.001
1 – Lowest	479,190	99.69	1,469	0.31		
2	344,130	99.56	1,534	0.44		
3	286,461	99.44	1,608	0.56		
4	170,386	99.31	1,190	0.69		
5 – Highest	179,670	99.18	1,490	0.82		
Social capital (cohort entry)	Median	IQR	Median	IQR		
Political trust	0.03	−0.05–0.11	0.04	−0.04–0.12	−2.9	<0.001
Welfare trust	−0.01	−0.16–0.11	−0.04	−0.17–0.10	4.9	<0.001
Personal trust	0.06	−0.26–0.44	−0.08	−0.36–0·34	19.7	<0.001

^a^Remainder of the complete case sample.

^b^Relative to the whole of Sweden.

^c^*χ*^2^ denotes a chi-squared test on a number of degrees of freedom (d.f.), denoted in brackets in this column. No adjustments for multiple comparisons are necessary.

### SPHC survey respondent representativeness and neighborhood-level trust

The majority of the 23,510 respondents to the 2002 SPHC were Swedish-born to two Swedish-born parents (72.20%; Extended Data Table 2). A total of 56.18% of respondents were female, and the median age of respondents was 48.0 years (interquartile range (IQR), 35.0–60.0). Respondents differed from the 2002 population of the Stockholm County catchment area on all measured characteristics in univariable comparisons. Following multivariable logistic regression, survey respondents were more likely to be women (odds ratio (OR) 1.29; 95% confidence interval (CI), 1.26–1.33), older (OR per year of age 1.01; 95% CI, 1.009–1.011) and from higher income quintiles than the general population (Extended Data Table 2). Notably, people with a foreign-born history were underrepresented in the SPHC survey, and this was most pronounced for those from Sub-Saharan Africa (OR 0.44; 95% CI, 0.37–0.53), North Africa and the Middle East (OR 0.59; 95% CI, 0.54–0.64) or Asia (OR 0.59; 95% CI, 0.52–0.68).

**Extended Data Fig. 1:**
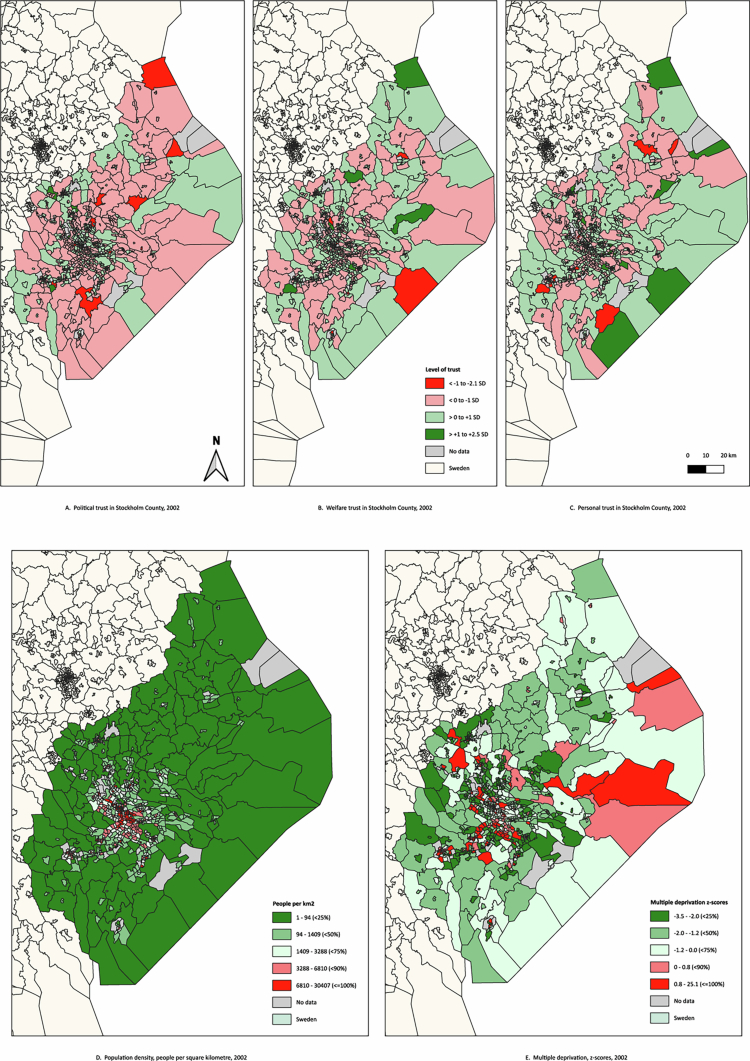
Variance in neighbourhood characteristics at SAMS-level in Stockholm County, 2002. Geographical distribution of neighbourhood characteristics at SAMS-level in Stockholm County, as estimated from 2002 data. Maps A-C show the distribution of median SAMS-level political, welfare and personal trust as rated by participants in the Stockholm Public Health Cohort survey. The choropleth maps in A-C are coded according to standard deviations from the mean level of trust in the whole county. Map D shows population density estimates and Map E shows socioeconomic deprivation estimates in Stockholm County in 2002. Both Map D and Map E are categorised at the 25th, 50th, 75th, 90th and 100th percentiles.

Following polychoric factor analysis of 13 SPHC survey items related to trust and social support, we identified three latent factors (Supplementary Fig. 1) which we termed political trust, welfare trust and personal trust (Extended Data Table 3), which we aggregated to the SAMS level to create median levels of neighborhood trust across all SAMS in Stockholm County in 2002 (Extended Data Fig. 1).

### Geographic variance in incidence and SAMS-level correlations

**Fig. 2:**
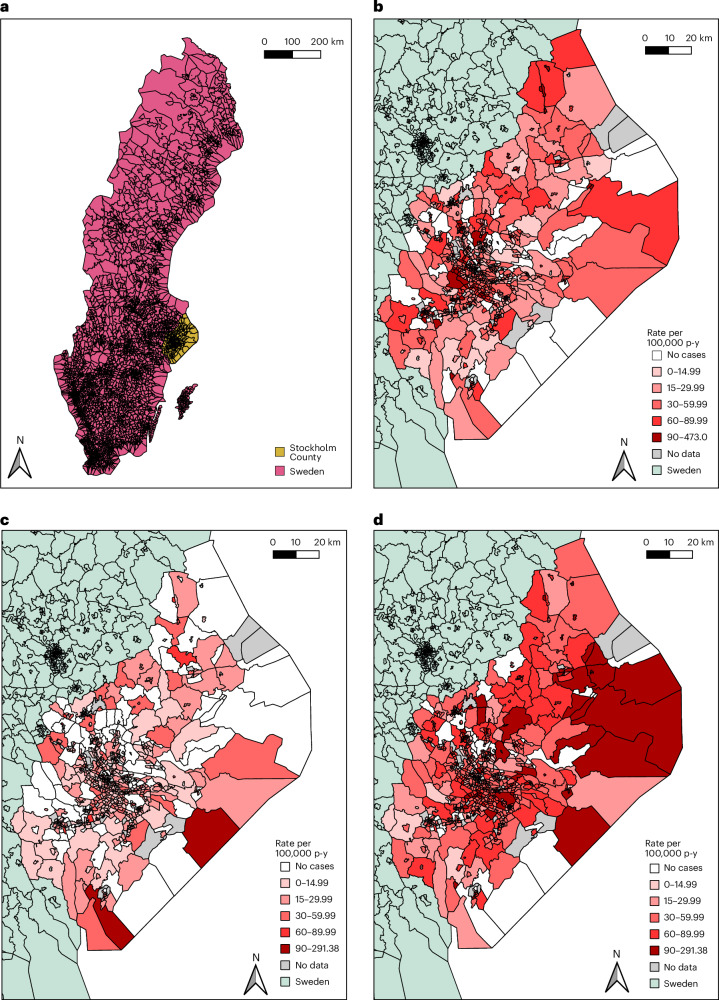
Map of Sweden and crude incidence of SMIs in Stockholm County, 2002–2016. **a**, SAMS geography in Sweden, and Stockholm County in relation to the rest of the country. **b**–**d**, Crude incidence per 100,000 person-years (p-y) of each SMI outcome in the Stockholm County catchment area between 2002 and 2016: non-affective psychoses (**b**), affective psychoses (**c**) and non-psychotic bipolar disorder (**d**). Category intervals across **b**–**d** are the same to aid relative interpretation. Visual inspection suggests rates of non-affective psychoses (**b**) are highest in SAMS within Stockholm city, and in several provincial towns in the county. No clear spatial gradient is evident for affective psychoses (**c**), whereas the highest rates of non-psychotic bipolar disorder (**d**) cluster in Stockholm city and towards the east of the county. Note that the maps do not take into account sample sizes, so precision around some SAMS-specific incidence rates may be low, particularly in more rural SAMS, distorting the visual impression of observed variation (as in **d**).

The crude incidence of non-affective psychotic disorders was 49.78 cases per 100,000 person-years (95% CI, 48.66–50.94; Table 2), higher than for affective psychotic disorders (15.94; 95% CI, 15.31–16.60), but lower than for bipolar disorder without psychosis (55.02; 95% CI, 53.84–56.24). Rates varied between SAMS neighborhoods across Stockholm County (Fig. 2b–d, Supplementary Fig. 2 and Supplementary Table 4), with a suggestion of higher rates of non-affective psychotic disorders (Fig. 2b) and bipolar disorder without psychosis (Fig. 2d) in SAMS in the city of Stockholm, as well as in coastal areas to the east of Stockholm County for the latter. No clear spatial distribution of affective psychotic disorders was evident (Fig. 2c).

**Table 2:** Incidence rates of SMI outcomes and correlation between incidence rates and SAMS-level variables at cohort entry^a^

Diagnostic outcome	*N*	Incidence rate (per 100,000 person-years)		Political trust^a^	Welfare trust^a^	Personal trust^a^	Population density^a^	Deprivation^a^
		Crude (95% CI)	SAMS level median (IQR)	*ρ* (*P* value)	*ρ* (*P* value)	*ρ* (*P* value)	*ρ* (*P* value)	*ρ* (*P* value)
Non-affective psychotic disorder	7 291	49.93 (48.79–51.09)	36.16 (19.45–62.38)	−0.07 0.0621	−0.03 0.4358	−0.32 **<0.0001**	0.28 **<0.0001**	0.48 **<0.0001**
Affective psychotic disorder	2 357	16.14 (15.50–16.81)	13.30 (0.00–21.67)	0.07 **0.0454**	0.06 0.0820	−0.13 **0.0002**	0.12 **0.0008**	0.10 **0.0028**
Bipolar disorder without psychosis	8 112	55.55 (54.35–56.77)	48.82 (30.06–70.93)	0.06 0.1015	−0.09 **0.0124**	−0.17 **<0.0001**	0.08 **0.0312**	0.14 **0.0001**
Correlation matrix								
Political trust				1				
Welfare trust				0.12 **0.0009**	1			
Personal trust				0.01 0.7914	0.13 **0.0002**	1		
Population density				−0.05 0.1783	−0.05 0.1329	−0.14 **0.0001**	1	
Deprivation				−0.06 0.1011	−0.01 0.8557	−0.22 **<0.0001**	0.23 **<0.0001**	1

^a^All correlations are based on two-sided tests. *P* values are reported to four significant digits, or *P* < 0.0001 if smaller. *P* < 0.05 values are shown in bold.

*ρ*, correlation; *N*, sample size.

Crude rates of all outcomes were negatively correlated with greater SAMS-level personal trust at cohort entry, being strongest for non-affective psychotic disorders (*ρ* = −0.32; *P* < 0.0001) and more moderate for affective psychotic disorders (*ρ* = −0.13; *P* = 0.0002) and bipolar disorder without psychosis (*ρ* = −0.17; *P* < 0.0001) (Table 2). Rates were also positively correlated with greater deprivation and population density, most substantively for non-affective psychotic disorders (Table 2).

Participants with any outcome of interest tended to live in neighborhoods with lower personal trust at cohort entry compared with the population at risk (all *P* < 0.0001; Table 1 and Supplementary Tables 2 and 3), but lived in neighborhoods with slightly higher welfare trust (*P* < 0.01; except affective psychotic disorders, *P* = 0.07). People with bipolar disorder without psychosis were also more likely to live in areas with higher political trust at cohort entry than the population at risk (*P* = 0.001; Supplementary Table 3).

### Multilevel survival modeling

In univariable models, a one-standard-deviation increase in exposure to time-varying neighborhood-level personal trust was associated with reduced incidence of all three outcomes (Table 3). This association persisted for non-affective psychotic disorders (hazard ratio (HR) 0.89; 95% CI, 0.83–0.96) and bipolar disorder without psychosis (HR 0.92; 95% CI, 0.85–0.99), with weaker evidence of a similar effect for affective psychotic disorders (HR 0.91; 95% CI, 0.82–1.01; *P* = 0.07), after full multivariable adjustment for age group, sex, their interaction, parental history of SMI, parental region of origin, family disposable income quintile at cohort entry, and time-varying deprivation quintiles, population density quintiles (except for affective psychotic disorders), own-group migrant density, and political and welfare trust. In these multivariable models, we also found evidence that greater neighborhood-level welfare trust was associated with a lower incidence of bipolar disorder without psychosis (HR 0.88; 95% CI, 0.79–0.99). No other associations with welfare or political trust were observed.

**Table 3:** Univariable and multivariable associations between SAMS-level social capital and incidence rates of selected psychiatric disorders in Stockholm County, 2002–2016

	Univariable		Final multivariable^a^		Wald *P* value^b^
	HR	95% CI	HR	95% CI	
Non-affective psychotic disorder					
Political trust	0.97	0.81–1.16	0.95	0.84–1.08	0.43
Welfare trust	0.77	0.65–0.91	1.00	0.90–1.12	0.97
Personal trust	0.55	0.50–0.60	0.89	0.83–0.96	0.002
Affective psychotic disorder					
Political trust	1.12	0.90–1.40	1.15	0.94–1.41	0.18
Welfare trust	0.92	0.77–1.10	1.00	0.84–1.18	0.99
Personal trust	0.72	0.66–0.79	0.91	0.82–1.01	0.07
Bipolar disorder without psychosis					
Political trust	1.17	1.01–1.35	1.10	0.97–1.27	0.16
Welfare trust	0.79	0.69–0.90	0.88	0.79–0.99	0.03
Personal trust	0.75	0.70–0.81	0.92	0.85–0.99	0.02

^a^Final multivariable multilevel survival model, adjusted for age group, sex, their interaction, parental history of SMI, parental region of origin, family disposable income quintile at cohort entry, and time-varying deprivation quintile, own-group migrant density and other social capital domains. For non-affective psychotic disorders, we also adjusted for time-varying population density quintile. See Supplementary Figs. 3 and 4.

^b^Two-sided.

We observed a strong statistical interaction (effect modification) on the multiplicative scale between neighborhood-level personal trust and parental region of origin for non-affective psychotic disorders (likelihood ratio test (LRT) *χ*^2^ *P* value on six degrees of freedom, 23.7; *P* = 0.0006; Table 4) and bipolar disorder without psychosis (LRT *χ*^2^, 30.6 (6); *P* < 0.0001), which operated similarly for both outcomes in our multivariable model. For example, for non-affective psychotic disorders, a one-standard-deviation increase in exposure to neighborhood personal trust was associated with a reduced incidence for participants of Swedish (HR 0.91; 95% CI, 0.84–0.98) or other European heritage (HR 0.80; 95% CI, 0.69–0.92) or whose parents originated from mixed regions of origin (HR 0.78; 95% CI, 0.66–0.92) (86.37% of this group had at least one Swedish-born parent, and a further 12.16% were born in Sweden to two parents from mixed European regions-of-origin; Supplementary Results, ‘Sample Characteristics: further details’ section in Supplementary Information and Extended Data Table 8). By contrast, a one-standard-deviation increase in exposure to neighborhood personal trust was associated with an increased incidence of non-affective psychotic disorders for those of Sub-Saharan African (HR 1.76; 95% CI, 1.00–3.08; *P* = 0.049) or North African and Middle Eastern (HR 1.46; 95% CI, 1.08–1.96) heritage. No statistically significant differences were observed for those of Asian heritage. We found no evidence of statistical interaction between parental region of origin and personal trust for the affective psychotic disorders (LRT *χ*^2^, 3.1 (6); *P* = 0.79), nor between parental region of origin and political or welfare trust for any outcome (Table 4).

**Table 4:** Effect modification of the association between SAMS-level social capital and incidence of selected psychiatric disorders, by parental region of origin, in Stockholm County, 2002–2016

	Cases	%	Final multivariable effect size for social capital on incidence^a^		LR test^b^
			HR	95% CI	*χ*^2^ (d.f.); *P* value
Non-affective psychotic disorder					
Political trust by parental region of origin			–	–	5.3 (6); 0.51
Welfare trust by parental region of origin			–	–	9.5 (6); 0.15
Personal trust by parental region of origin			–	–	23.7 (6); 0.0006
Sweden	5,162	70.8	0.91	0.84–0.98	
Other Europe	1,009	13.8	0.80	0.69–0.92	
Asia	19	0.3	1.58	0.65–3.84	
North Africa and Middle East	200	2.7	1.46	1.08–1.96	
Sub-Saharan Africa	74	1.0	1.76	1.00–3.08	
Mixed	784	10.8	0.78	0.66–0.92	
Other	43	0.6	1.15	0.59–2.26	
Affective psychotic disorder					
Political trust by parental region of origin			–	–	8.0 (6); 0.24
Welfare trust by parental region of origin			–	–	1.4 (6); 0.97
Personal trust by parental region of origin			–	–	3.1 (6); 0.79
Sweden	1,795	76.2	–	–	
Other Europe	262	11.1	–	–	
Asia	<5^c^	0.2	–	–	
North Africa and Middle East	50	2.1	–	–	
Sub-Saharan Africa	13	0.6	–	–	
Mixed	225	9.5	–	–	
Other	<10^c^	0.3	–	–	
Bipolar disorder without psychosis					
Political trust by parental region of origin			–	–	4.3 (6); 0.63
Welfare trust by parental region of origin			–	–	7.2 (6); 0.31
Personal trust by parental region of origin			–	–	30.6 (6); <0.0001
Sweden	6,386	78.7	0.86	0.80–0.94	
Other Europe	896	11.0	1.09	0.94–1.28	
Asia	14	0.2	0.69	0.22–2.19	
North Africa and Middle East	75	0.9	1.89	1.20–2.99	
Sub-Saharan Africa	11	0.1	5.05	1.50–16.99	
Mixed	705	8.7	1.12	0.94–1.33	
Other	25	0.3	1.23	0.51–2.93	

^a^Final multivariable multilevel survival model, adjusted for age group, sex, their interaction, parental history of SMI, parental region of origin (except where stratified results are presented), family disposable income quintile at cohort entry, and time-varying deprivation quintile, own-group migrant density and other social capital domains. For non-affective psychotic disorders, we also adjusted for the time-varying population density quintile. See Supplementary Figs. 3 and 4.

^b^Two-sided.

^c^Values suppressed due to possible risk of disclosure in cells where *n* < 5. The dashes represent cells where reporting results would not be applicable to the table (they are limited to HR and their 95% CI for the three outcomes for each of three measures of trust).

### Sensitivity analyses

**Extended Data Fig. 2:**
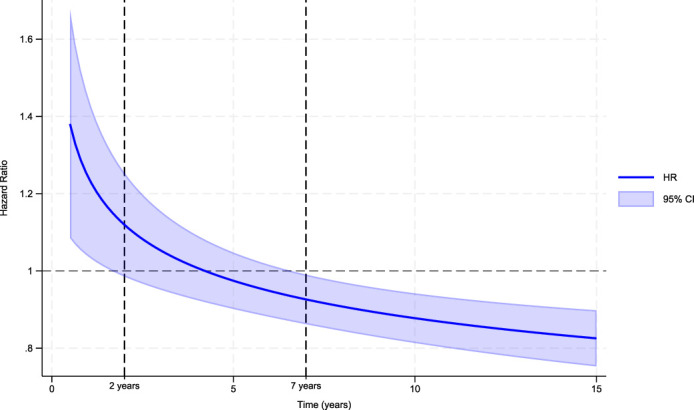
Departure from proportional hazards over the follow-up period between neighbourhood-level personal trust non-psychotic bipolar disorders. We found evidence of non-proportional hazards (LRT p < 0.0001) for the association between neighbourhood-level personal trust and non-psychotic bipolar disorder over time in our main multivariable model (from Table 3). This suggested that higher levels of neighbourhood-level personal trust were initially associated with increased rates of non-psychotic bipolar disorder (up until approximately two years into the follow-up period), but over time higher neighbourhood-level personal trust became associated with lower rates of non-psychotic bipolar disorder (becoming statistically significant about seven years into the follow-up period; see also Extended Data Table 7).

Findings across all outcomes remained similar after we regenerated neighborhood-level social capital scores and re-ran our analyses, following exclusion of 280 SPHC respondents who were diagnosed with an outcome of interest after the 2002 SPHC survey was conducted (Extended Data Table 4). The findings also remained consistent after excluding 106 SAMS areas where social capital scores were derived from fewer than five SPHC respondents in 2002 (Extended Data Table 5). In post hoc instrumental variable analyses, using median SAMS-level voter turnout in the 2002 Swedish County Council elections as an instrument for personal trust in the neighborhood, our results for the Swedish-born group and migrants from other European countries remained consistent with our main findings (Extended Data Table 6 and Supplementary Results), indicating a protective effect of neighborhood-level personal trust on the incidence of non-affective psychotic disorders and bipolar disorder without psychosis, as before. No evidence of this protective effect was observed for migrants from other countries, and in some cases (including for migrants from Sub-Saharan Africa and North Africa and the Middle East) there was evidence their rates remained elevated (Extended Data Table 6 and Supplementary Results), as before. Finally, although we found no evidence of departure from proportional hazards in the association between neighborhood-level personal trust and rates of non-affective psychotic disorder (LRT *P* = 0.24) or affective psychotic disorders (LRT *P* = 0.85), we did observe evidence of non-proportional hazards over time for bipolar disorder without psychosis (LRT *P* < 0.0001); here, greater neighborhood-level personal trust was associated with higher rates in the first two years of follow-up (that is, HR_1-year_ 1.24; 95% CI, 1.04–1.45; HR_2-year_ 1.19; 95% CI, 0.99–1.25; Extended Data Table 7), but became protective over the longer term, up to 15 years later (that is, HR_15-year_ 0.83; 95% CI, 0.75–0.89; Extended Data Table 7 and Extended Data Fig. 2).

## Discussion

### Principal findings

This longitudinal study finds protective effects of greater exposure to neighborhood-level personal trust on subsequent lower rates of severe mental illness (SMI), including non-affective psychotic disorders and bipolar disorder without psychosis, independent of individual- and area-level confounders. Importantly, our analyses show that these protective effects—disproportionately based on ratings of personal trust by people of Swedish-born heritage—only applied to that group and those of European or mixed (predominantly Swedish-European) heritage. By contrast, the same levels of personal trust increased the rates of these outcomes for those of African and Middle Eastern heritage. These results are consistent with our hypotheses, and empirically support the theory that apparent levels of bonding or relational social capital can simultaneously produce protective and harmful effects on mental health for different segments of the population, potentially dependent on perceived in- or outgroup membership.

### Interpretation

Our construct of personal trust aligns closely with theoretical conceptualizations of bonding or relational social capital^33,34^, with political and welfare trust aligning more closely with linking social capital (Extended Data Table 1)^35^. As such, our results extend cross-sectional evidence of a putatively protective association between neighborhood-level relational or bonding social capital and non-affective psychotic disorders^3,19–21^, by showing that such effects are present longitudinally for the majority Swedish-born population during 15 years of follow-up. These findings were also apparent for bipolar disorder without psychosis, although we found some evidence that the effect of personal trust was initially associated with higher rates of bipolar disorder without psychosis in the first two years of follow-up, before becoming associated with a protective effect on rates over the next 13 years. One possibility here is that, in the short term, more cohesive communities help members seek treatment, but protective effects emerge over the longer term (Extended Data Fig. 2 and Extended Data Table 7). Further empirical research on this issue is required.

The only previous longitudinal study on social capital and SMI, also conducted in Sweden, was restricted to a much shorter follow-up (two years), but also reported a protective effect of linking social capital (measured via higher voter turnout) on hospitalization risk for non-affective psychotic disorders, but not depression^26^. Other cross-sectional studies have also found an association between higher neighborhood-level voter participation and lower incidence of non-affective psychotic disorders^20,21^. In one of those, Kirkbride and colleagues^20^ theorized that voter turnout at local elections may be more closely aligned to bonding than linking social capital, encapsulating people’s willingness to invest in their community, something supported by our instrumental variable results in the present study (Extended Data Table 5). Our results for SMI are consistent with longitudinal research, including natural experiments^36,37^, on other mental health outcomes. These studies have found protective effects of both bonding and structural social capital on psychological distress^37^ and cognitive function^36^, respectively, two intermediary phenotypes that are both perturbed in people experiencing non-affective psychoses.

Our findings also support theoretical perspectives that certain forms of social capital—when exclusionary—may have negative outcomes for outgroups^16,38^, extending one cross-sectional study of social cohesion and non-affective psychotic disorders that observed similar findings^23^. Here we have found that Swedish-born participants of African and Middle Eastern heritage were at increased risk of non-affective psychotic disorders and bipolar disorder without psychosis in neighborhoods with greater levels of personal trust as disproportionately rated by the Swedish majority population. If considered as outgroups, these groups may have been unable to access the apparent levels of bonding or relational social capital measured in our study. From the perspective of intergroup contact theory^39^—a branch of social psychology that seeks to understand how conditions that govern intergroup contacts have positive or negative effects on prejudice—high levels of bonding or relational social capital perceived by the ingroup may provide conditions that uphold cycles of structural racism and psychosocial disempowerment, at the cost of poorer mental health, among other outcomes^40^, for outgroups with fewer opportunities to access, develop or maintain strong social ties. These maladaptive conditions have been shown recently to account for much of the excess risk of psychotic disorders experienced by people from ethnic minority and migrant backgrounds^14^. Future interdisciplinary research should explore these issues. They should also examine whether unbiased, group-specific measures of bonding or relational social capital do indeed exert protective effects for given groups, which would support our observations, and whether those that show greater own-group ethnic^24^ or migrant^25^ density are associated with lower rates of non-affective psychotic disorders. We also need to understand whether bridging social capital (Extended Data Table 1)—which could promote positive intergroup contact—is protective against risk of SMIs. Unfortunately, empirical measures of bridging social capital were unavailable in the present study. Nonetheless, work in East London has shown that increased ethnic integration (a prerequisite for bridging social capital) is associated with reduced rates of non-affective psychotic disorders in Black Caribbean communities^8^, and more relevant in that context than own-group ethnic density (a prerequisite for bonding or relational social capital).

If the epidemiological signals we have detected are causally relevant to the onset of major psychiatric conditions, this needs to correspond to plausible biopsychosocial mechanisms that trigger psychosis- or bipolar-related phenomena at the neurobiological level. There is growing neuroscientific evidence to support this possibility. For example, compared with non-migrants, migrant participants have been shown to have greater reactivity following stress challenges in brain regions including the perigenual anterior cingulate cortex and ventral striatum^41^, two brain regions that lie upstream and downstream of the amygdala, respectively. This connected region is critical to stress regulation, emotional conflict resolution and threat processing, which when disrupted may lead to the development of aberrant perception and beliefs^42^, two fundamental symptoms of psychosis. Experiential stress sensitivity, threat perception and aberrant salience have all been associated with greater psychosis liability, including sensitivity specific to outgroup stress^43^. Migrants also experience greater striatal dopamine release and synthesis capacity following exposure to stress compared with non-migrants^44^. McCutcheon et al.^45^ have also found that exposure to outgroup faces was associated with greater amygdala reactivity in both Black and White participants, but that for Black participants this effect was more pronounced for those living in communities with fewer Black residents, consistent with a buffering role of social capital on mental health^17^.

### Strengths and limitations

The longitudinal design, large sample, causally informed identification of confounders via directed acyclic graphs (DAGs; Supplementary Figs. 3 and 4), and treatment of both exposures and neighborhood-level confounders as time-varying strengthen the internal validity of our results. Diagnostic data were obtained from the Swedish national patient register, which provided almost complete coverage of inpatient settings and at least 80% coverage of outpatient settings in Sweden over the follow-up period^46^. The reliability and validity of psychotic disorder diagnoses in the national patient register^47^, as well as the hierarchical classification system we used to assign people to their SMI outcome^7,11^, have good apparent validity. The national patient register has near-complete coverage of secondary and emergency care providers in Sweden, and it is unlikely that people presenting with a first episode of an SMI would be treated solely in primary care. We used empirically derived social capital data at the small area level from a large, independent sample of neighborhood informants living in Stockholm County at the start of the follow-up period, and used polychoric factor analysis to provide valid estimates of the underlying factor structure of these constructs in the presence of ordinal data^48^. We also ensured our resultant factor structure was robust against overfitting (Methods)^49^. Finally, we conducted several sensitivity analyses to assess whether the potential for possible biases (including reverse causality, measurement error and endogeneity of the exposure via instrumental variable analyses) could have influenced our results.

Several limitations need acknowledgment. First, although we modeled social capital as time-varying, our constructs were only measured at cohort entry (2002) and were assumed to be constant within neighborhoods over time. Our treatment of social capital would thus have captured exposure changes as people moved neighborhoods within Stockholm County, but not absolute changes in social capital within neighborhoods or relative changes between neighborhoods over time. Although difficult to assess, this may have introduced bias if neighborhood social capital changed more quickly in certain neighborhoods over this period (for example, due to gentrification) and if this change was associated with subsequent incidence rates. Control for time-varying population density and deprivation did not suffer from this issue, as we were able to regenerate these values for all SAMS areas annually.

Second, our derived constructs of social capital were based on items related to trust in different individuals, groups and institutions that people may engage with in their daily lives (healthcare, welfare services, police, politicians, neighbors and peer networks), as asked in the SPHC. In our study, these mapped onto constructs we termed political, welfare and personal trust. The extent to which these correspond to theorized models of social capital (Extended Data Table 1) needs consideration. Most obviously, our constructs of political and welfare trust align to Szreter and Woolcock’s^35^ concept of linking social capital, whereas personal trust aligns to what Coleman^50^ and Putnam^33^ would consider bonding social capital or the idea of relational social capital. Our study warrants further replication, using validated measures of various forms of social capital^34^.

Third, deriving ecological measures of social capital by aggregating individual ratings does not guarantee reliable neighborhood estimates of the underlying constructs, even when individual ratings are reliable^51^. Although our social capital measures exhibited good individual-level reliability, this did not necessarily hold at the neighborhood level (Extended Data Table 3), particularly for measures of political and welfare trust, and caution in interpreting the results associated with these domains is warranted. Personal trust, on which our main findings are predicated, showed moderate neighborhood-level reliability, lending more confidence to the validity of these findings. Poorer neighborhood-level reliability may indicate a lack of consensus between respondents within neighborhoods, possibly evidenced by the presence of strong effect modification between personal trust and parental region of origin on two of our three outcomes. It may also have been the result of greater sampling error when responses are based on smaller samples, something we investigated but found little support for in sensitivity analyses.

Fourth, we acknowledge that our neighborhood definitions were based on administrative SAMS units, which may not correspond to ecologically meaningful communities as experienced by participants themselves. Nonetheless, SAMS were designed to maximize internal homogeneity with respect to housing type, date of construction and tenure form, and are relevant to understanding variation in social position in the population^52^, lending some credence to their validity here.

Fifth, although we were able to exclude prevalent cases that were diagnosed in Sweden after 1973 but before the start of our follow-up period (from 2002), we may have included some older prevalent cases in our study who would have been diagnosed before 1973 and then not again until after 2002. We believe this number would have been small and thus unlikely to have substantially biased our findings (Supplementary Methods).

Sixth, we were unable to study depression in this Article, as the national patient register is not linked to primary care, but our work warrants future studies on this and other mental health outcomes.

Finally, although we constructed DAGs to inform confounder selection, we are unable to infer causality from our results given the potential for unobserved confounding, most notably arising from imperfect control for possible genetic selection effects into lower social capital environments^53–56^, or the potentially causal role of cannabis use on psychosis risk^57^. Our DAGs suggested these issues preclude us from assuming a causal relationship between personal trust and our outcomes, unless strong assumptions hold (Supplementary Figs. 3 and 4 present a complete discussion). Briefly, to assume causality, we would require all genetic vulnerability to SMIs to be captured by our measure of parental history, which is unlikely^1^, and for there to be no effect of individual cannabis use on neighborhood-level estimates of social capital in SPHC survey respondents. The latter may hold if cannabis use is low in the general population, as seems the case in Sweden, with an estimated one-month prevalence of 1.4% in 2018^58^. Further research is required to strengthen observational studies against these strong caveats.

### Policy implications

If valid, our results suggest that promoting better bonding or relational social capital for the majority population is unlikely to be protective against major psychiatric conditions for all, and for some groups it may exacerbate risk, widening inequalities. We suggest that public policy could adopt strategies that promote positive intergroup contact as the grounds for developing and sustaining inclusive social capital that connects across different sociodemographic groups (that is, bridging social capital). Ingroup trust may extend to outgroups when grounds of cooperation can be established^59^, so facilitating bridging ties between migrant and Swedish groups could be prioritized in line with a group inclusion model^60,61^. Where possible, bottom–up approaches that support residents’ active involvement in redesigning services and systems that improve community safety and provide opportunities for employment, connectivity and collective action may provide the foundation for better social capital^62^. Interventions that promote integration include ‘buddy schemes’, which match migrant families with local families, community consultation to identify the needs and priorities of different groups, and the promotion of inclusive, culturally diverse services, events and activities that engage and empower both migrant and non-migrant communities alike^60,63^. Similar schemes already operate in Sweden^64^, but will require careful evaluation to understand whether such complex interventions may ameliorate the risk of severe psychiatric problems in different population groups.

## Methods

### Study design, setting and participants

We used the national Register of the Total Population to define cohort participants who were born in Sweden and who lived in Stockholm County while aged 14–64 years from 1 January 2002 to 31 December 2016. We geocoded participants to their residential neighborhoods during follow-up, based on the smallest administrative geography in Sweden, known as SAMS areas (median population in Stockholm County in 2002, 1,332; IQR, 572–2,566; Supplementary Methods provides further details). We restricted our cohort to participants who, at cohort entry, were resident in a subset of 813 of these 890 SAMS (henceforth, the ‘Stockholm County catchment’) for which we had SPHC data on social capital (‘Exposures’ section) and relevant area-level covariate data (‘Confounders’ section). Cohort entry was from 1 January 2002 (if resident in the Stockholm County catchment and aged 14–64 years on this date), from their 14th birthday (if later, and resident in the catchment on this date) or from their earliest date of residence in the catchment (if later, and aged 14–64 years). Cohort exit was the first date of an outcome of interest (below), 65th birthday, emigration from Sweden, change in registered address to a SAMS outside of the catchment (Supplementary Methods), death or 31 December 2016, whichever was sooner. We excluded people who were diagnosed with any outcome of interest before the beginning of follow-up. Informed consent for registry-based research is not applicable.

### Data

#### Outcome measures

Our primary outcome was first diagnosis of an International Classification of Diseases, tenth revision (ICD-10) non-affective psychotic disorder (F20–29), as recorded in the National Patient Register, which has included psychiatric diagnoses since 1973, near-complete coverage of inpatient settings since 1987 and outpatient settings since 2001. We also included affective psychotic disorders (F30.2, F31.2, F31.5, F32.3, F33.3) and bipolar disorder without psychosis (F30.X, F31.X, excluding the aforementioned codes) as secondary outcomes (Supplementary Methods provides further details).

#### Exposures

We linked cohort participants to empirically derived domains of social capital in each SAMS in the Stockholm County catchment, independently rated from a random sample of 23,510 people who gave informed consent to take part in the 2002 SPHC survey (Supplementary Methods)^31^. We included 14 items related to social capital, including nine items related to trust in state-provided services and democracy, four items related to social support and trust in the residential area and one item on whether respondents voted in the 2002 Swedish elections (yes/no). All item responses (except voting) were rated on a Likert scale from 1 (yes, always) to 4 (no, never), and an option to state ‘no opinion’, which we considered a missing data problem^65^, and handled via multiple imputation by chained equations (Supplementary Methods). A total of 15,519 SPHC respondents (66.0%) had at least one item treated as missing (Supplementary Fig. 5). Item-level missingness varied from 0% to 45% (Supplementary Table 5).

Following multiple imputation, we conducted polychoric factor analysis on the imputed dataset, which led to the identification of three social capital factors, which we termed political trust, welfare trust and personal trust (Extended Data Table 3 and Supplementary Fig. 1). We estimated median neighborhood-level factor scores for these three exposures based on individual factor scores for all SPHC respondents reporting on the same SAMS neighborhood. We performed a Box-Cox transformation on median factor scores to handle skew, and *z*-standardized scores to have a mean of zero and standard deviation of one. We estimated individual- and SAMS-level reliability in our social capital measures using established methods (Supplementary Methods and Extended Data Table 3), and ran k-fold cross-validation procedures in sensitivity analyses to evaluate the robustness of our factor structure to overfitting (Supplementary Methods and Supplementary Table 6)^49^. This indicated that the underlying factor structure was robust, with very low error estimates between our original and k-fold-derived predicted factor scores.

#### Confounders

We constructed DAGs (Supplementary Figs. 3 and 4 and Supplementary Methods) to control for several relevant a priori confounders, including biological sex at birth (male, female), age group (14–19 years, then five-year age bands until 60–64 years), history of any of the aforementioned psychiatric outcomes in a biological parent, migrant status (Swedish-born to two Swedish-born parents, or children of migrants), parental region of origin (Swedish-born, Other Europe, Asia, North Africa and Middle East, Sub-Saharan Africa, Mixed and Other), disposable family income quintile at cohort entry, and time-varying deprivation quintile and, for non-affective psychotic disorders only, time-varying population density quintile (full details are provided in Supplementary Methods).

### Statistical analyses

We conducted appropriate tests (*χ*^2^, Mann–Whitney U-test, Kruskal–Wallis and Spearman’s correlations) to examine descriptive relationships between outcomes, exposures and confounders, and missing data patterns. We also examined the representativeness of SPHC survey respondents to the total population of the catchment in 2002, via descriptive statistics and multilevel, multivariable logistic regression. We conducted multilevel (random intercepts) parametric survival models with a Weibull distribution to account for the hierarchical nature of our dataset and to model time-varying covariates (age, social capital, deprivation, population density) over the follow-up period (Supplementary Methods). Modeling proceeded as follows for each outcome. First, we quantified the proportion of variance (in the outcome hazard rate) attributable to the SAMS level in null and individual-level adjusted (age group, sex, their interaction, parental history of our psychiatric outcomes, family disposable income quintile) and fully adjusted multivariable models, estimated by the random intercepts variance parameter (*σ*^2^) (Supplementary Table 4). Second, we fitted univariable models between each outcome and social capital exposure. Third, we fitted multivariable models, controlling for confounders identified via our DAGs (see above). Fourth, we tested for effect modification between each social capital exposure and our outcomes by parental region of origin, assessed via an LRT between nested models with and without the interaction term. We reported unadjusted and adjusted HR and 95% CI for all measures of effect. Finally, we performed four sensitivity analyses to consider the impact on our results of (1) excluding social capital responses from SPHC respondents diagnosed with an outcome of interest after 2002; (2) excluding SAMS where social capital responses were based on fewer than five SPHC respondents; (3) possible endogeneity in social capital as an exposure^66^, by fitting two novel instrumental variable analysis methods developed for survival data^67^; and (4) to check for potential departure from proportional hazards in our models (Supplementary Methods). We conducted complete case analyses given the small proportion of missing data in the cohort. All modeling was conducted in Stata version 18.2.

### Ethical approval

This study was approved by the Stockholm Regional Ethical Review Board (2010/1185-31/5) and the UCL Research Ethics Committee (21019/001).

### Reporting summary

Further information on research design is available in the Nature Portfolio Reporting Summary linked to this article.

## Supplementary information

Supplementary Information Supplementary methods, Results, Figs. 1–5, Tables 1–7 and references.

Reporting Summary

## Figures and Tables

**Extended Data Table 1:**
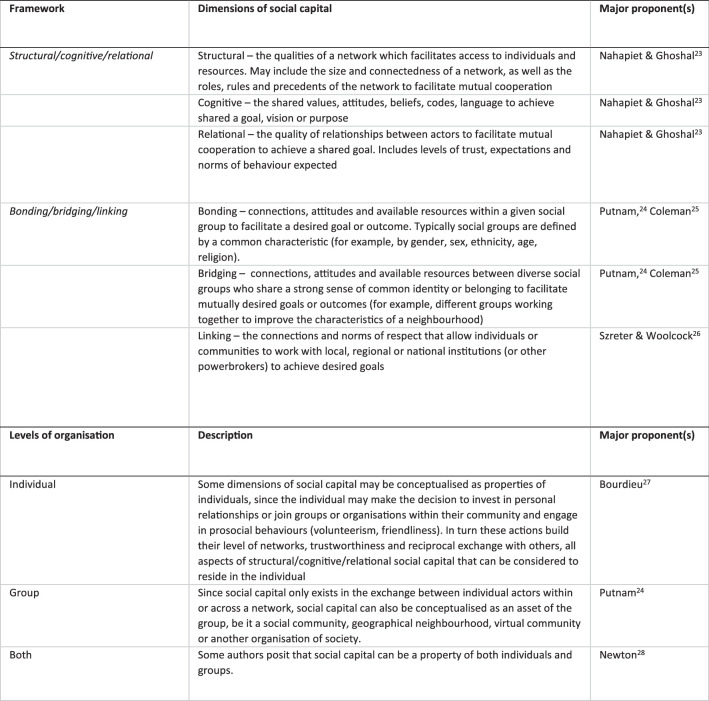
Commonly theorised frameworks and levels of organisation for social capital

**Extended Data Table 2:**
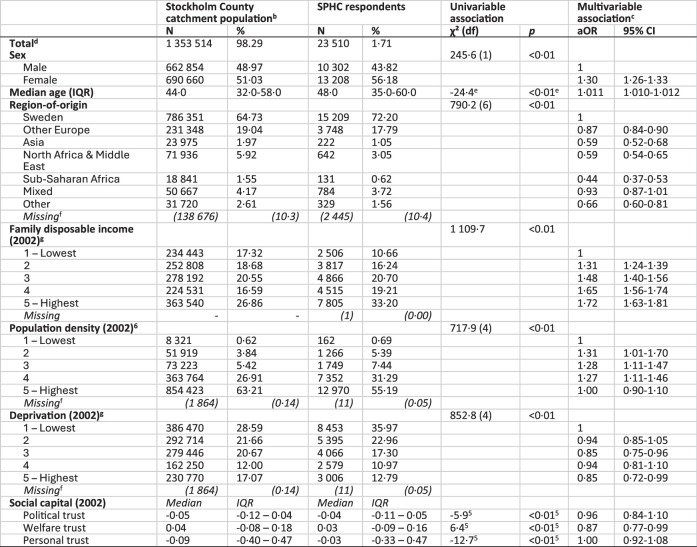
Representativeness of SPHC respondents to Stockholm County catchment area population in 2002^a^

IQR: interquartile range; df: degrees of freedom; aOR: adjusted odds ratio; CI: confidence interval

^a^Excluding people diagnosed with SMI before 2002. Catchment area restricted to 813 of 890 SAMS in Stockholm County with valid SPHC social capital data in 2002 and available deprivation data.

^b^Not including the SPHC sample. Estimated from the Register of the Total Population [RTP] in 2002, aged 18-84.

^c^Adjusted for all variables in table from a complete case analysis (N = 1 237 161; 87·9%) using multilevel logistic regression

^d^Row percentage; remainder of table presents column percentages

^e^Mann-Whitney U-test

^f^Missing percentage as a total of whole sample, not included in reported univariable statistics

^g^Relative to whole of Sweden

**Extended Data Table 3:**
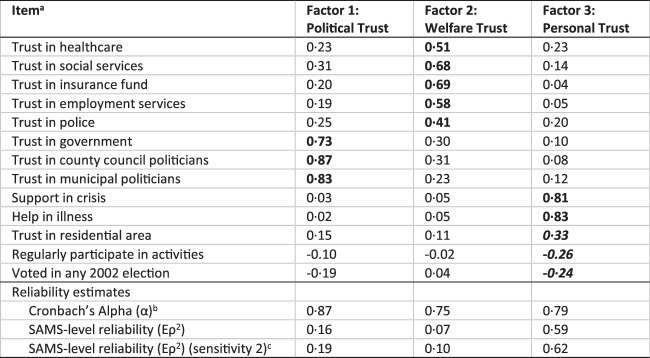
Rotated factor loadings of SPHC items on Social Capital sub-domains

Loadings ≥0.40 in **bold**. Strongest loadings for items with no factor loadings ≥0.40 shown in ***bold italic***.

^a^Trust in parliament was dropped from the factor analysis due to high collinearity with trust in government (ρ = 0·86), as the former had higher levels of missingness (See Supplementary Table 5).

^b^Based on items with factor loadings ≥0.40

^c^When restricted to SAMS with 5 or more SPHC respondents in Stockholm County – see second sensitivity analysis

**Extended Data Table 4:**
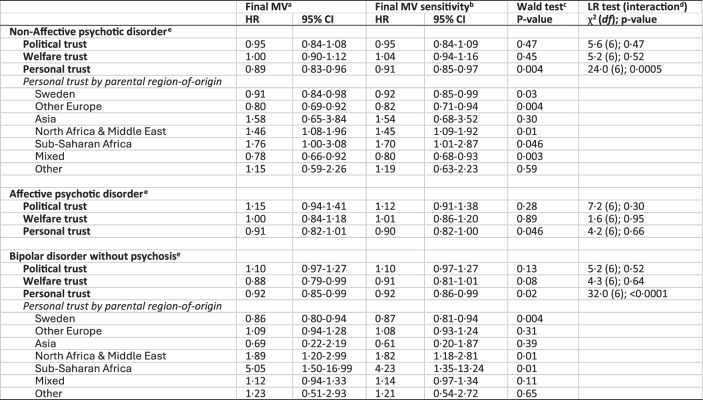
Sensitivity analysis of the longitudinal association between social capital and incidence of severe mental illnesses in final multivariable models, after regenerating social capital scores excluding 280 SPHC respondents in 2002 later diagnosed with SMI

MV: Multivariable; HR: Hazard ratio; CI: confidence interval: LR: likelihood ratio; df: degrees of freedom

^a^From Table 3 (main effects of trust variables on risk) or Table 4 (region-specific personal trust estimates)

^b^Excluding social capital scores from N = 280 of 23 510 (1·2%) respondents who took part in the SPHC survey in 2002, but who later became diagnosed with SMI

^c^Wald p-value for HR associated with parameter of interest

^d^Likelihood ratio test for statistical interaction between each *trust* measure and region-of-origin. Stratified estimates provided where LRT p < 0·05. For equivalent p-values for the final MV model, see Table 4

^e^Adjusted for age group, sex, their interaction, parental history of SMI, parental region-of-origin (except where stratified results are presented), family disposable income quintile at cohort entry, and time-varying deprivation quintile, own-group migrant density, and other social capital domains. For non-affective psychotic disorders, we additionally adjusted for time-varying population density quintile. See Supplementary Figs. 3 and 4

**Extended Data Table 5:**
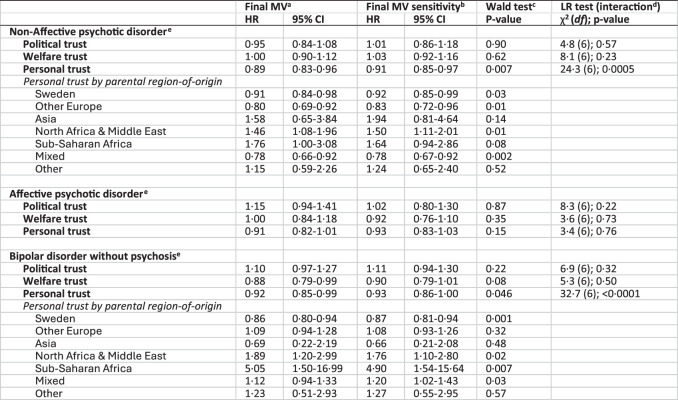
Sensitivity analysis of the association between social capital and incidence of severe mental illnesses in final multivariable models, after excluding participants living in SAMS during follow-up with less than 5 SPHC respondents in 2002

MV: Multivariable; HR: Hazard ratio; CI: confidence interval: LR: likelihood ratio; df: degrees of freedom

^a^From Table 3 (main effects of trust variables on hazard ratios) or Table 4 (region-specific personal trust estimates)

^b^Excluding 106 SAMS where less than 5 people contributed to SPHC social capital responses in 2002. 67 005 cohort participants in the complete case sample were excluded who had lived in these SAMS at some point during the follow-up period. This excluded 251 participants with non-affective psychotic disorders (3·4%), 79 participants with affective psychotic disorders (3·4%) and 390 participants with non-psychotic bipolar disorder (4·8%) over 747 262·3 person-years (5·1%)

^c^Wald p-value for HR associated with parameter of interest

^d^Likelihood ratio test for statistical interaction between each *trust* measure and region-of-origin. Stratified estimates provided where LRT p < 0·05. For equivalent p-values for the final MV model, see Table 4

^e^Adjusted for age group, sex, their interaction, parental history of SMI, parental region-of-origin (except where stratified results are presented), family disposable income quintile at cohort entry, and time-varying deprivation quintile, own-group migrant density, and other social capital domains. For non-affective psychotic disorders, we additionally adjusted for time-varying population density quintile. See Supplementary Figs. 3 and 4

**Extended Data Table 6:**
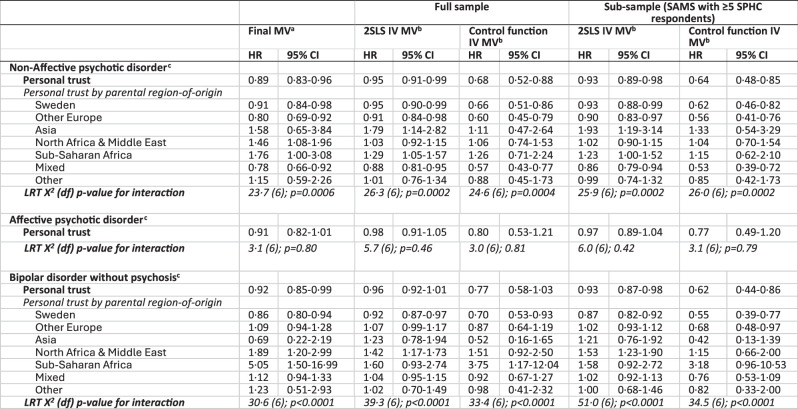
Sensitivity analyses of instrumental variable analyses using SAMS-level 2002 voter turnout at county council elections as an IV for personal trust in multilevel Cox proportional survival models

SPHC: Stockholm Public Health Cohort; MV: Multivariable; IV; Instrumental Variable; HR: Hazard ratio; CI: confidence interval: LRT: likelihood ratio test; df: degrees of freedom

^a^From Table 3 (main effects) or Table 4 (parental region-specific effects)

^b^See Supplementary Methods - Statistical analyses: sensitivity analyses

^c^Adjusted for age group, sex, their interaction, parental history of SMI, parental region-of-origin (except where stratified results are presented), family disposable income quintile at cohort entry, and time-varying deprivation quintile, own-group migrant density, and other social capital domains. For non-affective psychotic disorders, we additionally adjusted for time-varying population density quintile. See Supplementary Figs. 3 and 4

**Extended Data Table 7:**
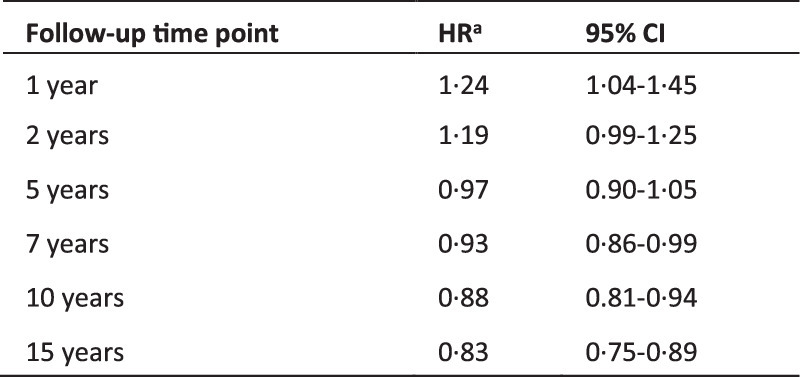
Hazard ratios of the association between neighbourhood-level personal trust and non-psychotic bipolar disorder at selected years of follow-up

HR: Hazard Ratio; 95% CI: 95% confidence interval

^a^Adjusted for all covariates reported in final multivariable model from Table 3

**Extended Data Table 8:**
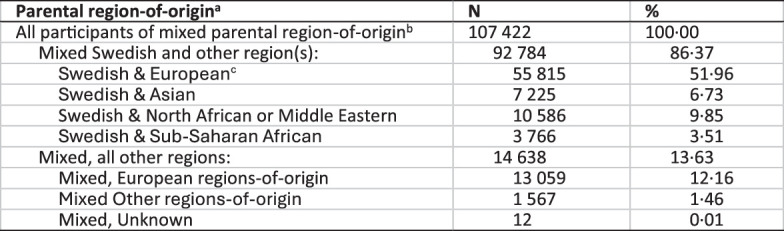
Further details of parental region-of-origin in participants of mixed parental heritage

^a^Based on biological mother and father’s birthplaces as recorded in the Register of the Total Population

^b^Participants in the cohort, see Table 1

^c^Including Russia

## Data Availability

Data for this study are available via controlled access due to ethical and legal issues surrounding the use of linked Swedish registry data and Stockholm Public Health Cohort data for research. These datasets are available via controlled access, and parties interested in using the data should contact Statistics Sweden (https://www.scb.se/en/) or the Swedish National Data Service (https://snd.gu.se/en/catalogue/study/ext0171).

## Code availability

The study protocol, analysis plan, statistical code and outputs are available as open access resources on a preprint server (10.31234/osf.io/269rx).
