# Supplementary material for: Longitudinal association between neighborhood-level social capital and incidence of major psychiatric disorders in a cohort of 1.4 million people in Sweden
Source: Nat Ment Health. 2025 Oct 20;3(11):1425–37. doi: 10.1038/s44220-025-00518-z (PMC12589101; doi:10.1038/s44220-025-00518-z)
Supplement: Supplementary file 1 — Supplementary methods, Results, Figs. 1–5, Tables 1–7 and references. [file 44220_2025_518_MOESM1_ESM.pdf]

nature mental health

Supplementary information

https://doi.org/10.1038/s44220-025-00518-z

Longitudinal association between neighborhood-level social capital and incidence of major psychiatric disorders in a cohort of 1.4 million people in Sweden

In the format provided by the authors and unedited

Table of Contents

Supplementary methods 2
Study design, setting and participants: further details 2
Outcome measures: further details 2
Exposures: further details 3
Statistical analyses: further details 8
Supplementary Results 11
SPHC survey respondent representativeness: further details 11
Sample characteristics: further details 11
Geographic variance in incidence and SAMS-level correlation: further details 12
Instrumental variable results: further details 12
Supplementary Figures 14
Supplementary Tables 21
Supplementary references 30

Supplementary methods

Study design, setting and participants: further details

SAMS are the smallest administrative unit available on which Swedish register data are disaggregated. In 2002, there were 8 945 SAMS in Sweden with a median population size of 707 people (interquartile range [IQR]: 306-1 316), of which 890 SAMS formed the region of Stockholm County (median population: 1 332; IQR: 572-2 566). We excluded 77 SAMS from our catchment area due to an absence of social capital data from the Stockholm County Public Health cohort (N=70) or for which deprivation could not be estimated (N=7), leaving 813 SAMS in the present catchment area. In 2002, 5 469 people aged 14-64 years lived in the 77 excluded SAMS, from a total of 1 245 159 people aged 14-64 years in all of Stockholm County; our catchment area thus covered approximately 99·6% of the population at-risk of Stockholm County. SAMS are designed to be stable over time, with boundaries designed to maximise homogeneity in housing type, period and tenure form.[1] The SAMS in which participants are living on 31 December each year are recorded for all participants in the Register of the Total Population. We used these SAMS codes to denote a persons residential address in any given year of interest, and to identify residential moves between SAMS neighbourhoods in any two consecutive years (also see "Statistical Analyses: further details", below).

Outcome measures: further details

We censored case participants at their first recorded date of any diagnosis of an outcome of interest during the follow-up period (i.e. NAPD, APD or NP-BPD). However, recognising the evolution of symptoms potentially captured by multiple diagnoses in the NPR over time, we used a previously validated hierarchical classification system[2] to assign participants to one of the three outcomes above. Here, any record of a diagnosis of NAPD superseded APD, which in turn superseded NP-BPD.

We excluded all people diagnosed with an outcome of interest before the follow-up date of our study (1 January 2002) since the National Patient Register began in 1973. This minimised the inclusion of prevalent rather than incident cases in our follow-up period. Since members of our cohort aged 44-64 in 2002 could have been diagnosed with an outcome of interest prior to 1973 (from their 14th birthday between 1952-1972) that was not recorded in the NPR, we could have erroneously included some prevalent cases in this age range in our case sample. Nonetheless, we consider this number would have been very small as this would have been a subsample of the cohort, diagnosed between 1952 and 1972, and then not diagnosed again until after 1 January 2002.

Exposures: further details

Our primary exposure variables were empirically-derived domains of social capital in each SAMS in Stockholm County, estimated from the 2002 wave of the SPHC.[3] The SPHC is a repeated-wave cross-sectional and longitudinal public health survey of up to 50 000 area-stratified randomly selected adults every 4 years, beginning in 2002. In 2002, participants aged 18-84 years old were recruited via a pre-notification letter, followed by a postal survey and 3 reminders with an additional survey. This led to 31 182 (62.3%) participants providing responses to the survey (Supplementary Fig. 5). Unfortunately, due to an administrative error, these participants were only retrospectively consented to take part in the survey at their first follow-up in 2007, leading to usable data from 23 771 eligible respondents (47·5% of original invitees, 76·2% of baseline respondents). From this sample, we excluded 261 respondents who either had an SMI diagnosis in the NPR prior to 2002 (N=155; 0·7% of participants providing eligible responses), or who had provided responses to the survey whilst resident in a SAMS outside of Stockholm County (N=106; 0·5%). This led to a final sample of 23 510 eligible respondents (47·0% of the original invitees, 75·4% of all respondents) in the SPHC, who provided responses to 14 items about social capital in Stockholm County in 2002. These included 9 items related to trust in state-provided services and democracy (healthcare, social services, insurance providers, employment services, police, parliament, government, county council politicians and municipal politicians); 4 items related to social support and trust, including support in a crisis, help in illness, involvement in community activities and trust in the residential area, and; one item on whether respondents voted in the 2002 Swedish elections (yes/no).

All responses (except voting participation) were recorded on a Likert scale from 1 (Yes, always) to 4 (No, never), with an additional fifth option to state "no opinion". We conceptualised "no opinion" responses as a form of missing data, where the true opinion of the respondent is unknown.[4] Provided that the data structure underlying missingness is missing-at-random [MAR], multiple imputation can be used to recover likely missing values. Under the MAR assumption, it has been shown that "no opinion" responses can be imputed to recover unbiased estimates of the missing items.[4] It has also been shown that missing survey response data vary strongly by sociodemographic characteristics.[5] By including a range of sociodemographic characteristics in our multiple imputation models we increased the probability that the assumption of MAR is satisfied. This method of handling "No opinion" responses is preferred to other common methods to handle such responses, including recoding these to be a "middle" response or complete case analysis, which can lead to biased estimates of the underlying constructs.

We performed multiple imputation by chained equations [MICE] to impute estimates for missing or "no opinion" responses to these 14 social capital items and one further auxiliary variable used in our imputation models (respondent socioeconomic status in 2002), which also contained missing values. We followed reporting guidelines for multiple imputation by Sterne et al.[6] Recent research has suggested that MICE can lead to unbiased imputations even in the presence of substantial (i.e. greater than 90%) missing data.[7] We included all 14 social capital items and several auxiliary sociodemographic variables in our imputation model, including age, sex, socioeconomic status and relationship status (married/civil partnership, single, divorced/widowed/separated), as recorded in the 2002 SPHC survey, as well as SAMS-level population density and deprivation quintiles in 2002, linked from our derived measures of these confounders from other register data, as described in our methods. There was evidence that these auxiliary variables were strongly correlated with the missing data pattern for most social capital item responses in the SPHC (Supplementary Table 5), particularly "trust" items; missing data patterns for items relating to support, help and participation in activities were in generally less strongly associated with sociodemographic patterns (Supplementary Table 5).

All variables requiring imputation were Likert-type responses, and so we used ordinal logistic regression models in the imputation procedure to impute missing values. After discarding 10 "burn-in" imputation sets, we ran 20 further imputations from a randomly generated set seed point. For each imputed dataset, we then conducted polychoric factor analysis, an extension of exploratory factor analysis for continuous variables, in the presence of ordinal data. This ordinal data is hypothesised to represent normally distributed continuous data, and polychoric factor analysis allows for the correct estimation of the correlation between these variables required for the eventual estimation of latent factors. Due to convergence issues caused by a high degree of collinearity between two social capital items (trust in parliament, trust in government; ρ=0·86), we omitted one of these variables ("trust in parliament") from polychoric covariance estimation to achieve convergence. "Trust in parliament" was dropped over "trust in government" due to a higher proportion of missing data (see Supplementary Table 5).

To conduct polychoric factor analysis on imputed data, we first estimated and retained the polychoric covariance matrix for each imputed dataset. We then estimated the mean covariance matrix across the 20 imputed datasets, and ran a factor analysis on this matrix. We inspected Kaiser-Meyer-Olkin [KMO] statistics to identify social capital items with poor sampling adequacy, that is, items whose variance might not be attributable to the underlying latent structure in the dataset. Item-level KMO was above 0·60 for all items, with a global KMO of 0·80 indicating adequate sampling. We performed

an oblique rotation (using the Oblimin method) to optimise factor loadings and allow factors to correlate. One item loaded poorly onto all factors (participation in community activities, <0·30) and so was omitted from the factor analysis, which we re-ran on the remaining 12 items. A screeplot of the resultant factor analysis (Supplementary Fig. 1) suggested a three-factor solution provided optimal fit of the data. Oblimin rotation confirmed most items loaded strongly (>0·40) onto a single factor, with two items (trust in social services, trust in county council politicians) loading moderately (>0·30) onto two factors (Extended Data Table 3).[8]

We interpreted the three factors as indicators of *political trust*, *welfare trust* and *personal trust*, as described in the main manuscript. We predicted factor scores for each SPHC respondent across the 20 imputed datasets, and derived their mean factor scores across these imputations. Given some skew in mean factor scores, we performed natural logarithmic transformations for each factor. We then z-standardised and reverse coded these factor scores, such that responses had a mean of zero and standard deviation of one, where higher scores indicated higher social capital. From this, we estimated the median social capital score for each factor for each SAMS in Stockholm County, based on SPHC respondents' SAMS residence at the time of the survey in 2002. We linked these median SAMS-level factor scores to each SAMS that our cohort participants had resided in during each year of follow-up to model change in exposure to social capital over the follow-up period (i.e. when moving to a new part of Stockholm County during follow-up).

We also estimated the individual- and SAMS-level reliability of our derived factors. At the individual-level, all three factors showed good internal consistency / reliability with Cronbach's alpha values ranging from 0·75 to 0·87 (Extended Data Table 3). Nonetheless, if there is considerable within-neighbourhood variability in social capital responses, aggregated social capital scores at the SAMS-level may not be reliable (i.e. may not accurately represent the true level of social capital experienced by all people living within that neighbourhood). It has been shown that aggregate reliability (i.e. here, reliability in SAMS-level median social capital scores) can be estimated using generalizability theory via estimation of the generalizability (or G) coefficient.[8] This estimates the degree of variance in responses within neighbourhoods as a proportion of the degree of variance in responses in the dataset, taking into account the number of respondents (to the SPHC survey) per neighbourhood. We estimated the G-coefficient ($E\rho^2$, Extended Data Table 3) for each social capital exposure in both our main analyses, and when we restricted our analyses to neighbourhoods with more than five SPHC respondents per SAMS providing information on social capital (as part of our sensitivity analyses; see methods). The G-coefficient can be interpreted in a similar way to Cronbach's alpha, with high values

indicative of good reliability at the neighbourhood level. Our data suggested that our neighbourhood-level measures of political ($E\rho^2$=0·17) and welfare ($E\rho^2$=0·05) capital had very low SAMS-level reliability, although neighbourhood-level personal trust showed greater (and moderate) reliability ($E\rho^2$=0·57). These values became higher (i.e. $E\rho^2_{\text{personal trust}}$=0·61) when restricted to SAMS with 5 or more SPHC respondents as part of our sensitivity analyses. We discuss the implications and limitation of these reliability estimates in the discussion section of the paper.

Finally, we inspected the robustness of our empirical factor structure to the potential issue of overfitting,[9] by using k-fold cross-validation in sensitivity analyses, and repeating the above procedure. To do so, we first randomly split the 23 510 participants in the SPHC survey into 10 bins (k=10) in the multiply imputed sample, such that the ith individual was allocated to the (same) k^{th} bin across all imputation samples. Next, we re-ran polychoric factor analysis in the k-1 training sample (90% of the sample), and predicted and retained factor scores in the kth testing sample. We repeated this procedure until all k bins had been included as the testing sample, and reconstructed predicted factor scores for the full sample obtained via this cross-validation methodology. We estimated the root mean square error and mean absolute square error of the differences between the predicted factor scores from the full sample and from the k-fold procedure to estimate the robustness of our factor structure to overfitting (Supplementary Table 6).

Confounders: further details

We used directed acyclic graphs (DAGs; Supplementary Fig. 3 and 4) to identify the minimum necessary set of confounding variables to control for which would permit valid causal inferences of the relationship between exposure (here, one of three social capital measure) and outcome (here, one of three psychiatric outcomes) to be made. We constructed separate DAGs for NAPD and APD/NP-BPD, as there is little evidence that the latter affective outcomes are associated with population density,[10] which was omitted as a confounder in these DAGs (Supplementary Fig. 4). For both DAGs, we acknowledged the potential unobserved confounding effect of genetic factors and cannabis use, for which data were not available in the Swedish registers. Failure to control for these variables has the potential to render any observed associations between our exposures and outcomes non-causal; full consideration of these issues and their likely effect on the associations observed in this study is provided in the discussion and figure legends (Supplementary Fig. 3 and 4).

Biological parental history of any psychiatric disorder was recorded via linkage to the multigenerational register and NPR, using the same ICD-10 codes as described for our main outcomes

of interest (see methods), and their ICD-8/9 equivalents (295.X, 296.X) for diagnoses in the NPR made between 1973-1996.

We classified participants' migrant status as either Swedish-born to Swedish-born parents or Swedish-born to at least one parent born outside of Sweden (henceforth, children of migrants), consistent with previous research.[2,11] We coded children of migrants according to their parental birthplaces, linking data held in the Register of the Total Population [RTP], Multigenerational Register and the immigration/emigration register, "STATIV". Parental region-of-origin was re-categorised as follows, based on both parents' country of birth: Sweden, Other Europe (Nordic, European), Asia, North Africa & Middle East, Sub-Saharan Africa, Mixed (Swedish-born to one Swedish and one foreign-born parent, or two foreign-born parents from different regions), and "other" (Oceania, North America, South America).

Disposable family income quintile in the year of cohort entry was estimated via linkage to the Longitudinal Integration Database for Health Insurance and Labour Market Studies [LISA] register. Disposable family income takes into account receipts from all sources including income, capital and welfare offset against annualised living costs according to household composition. For each year of cohort entry, we created disposable family income quintiles relative to all adults aged 16 or over in Sweden, to implicitly account for possible inflation over the study period. Because disposable family income is only recorded in the LISA register for people aged 16 years and over, those participants who entered our cohort before this age (i.e. from their 14th birthday; N=321 673 (2·05%) of the total analytic cohort; Fig. 1) were linked to their parents via the multigenerational register to obtain their correct level of family disposable income at cohort entry. Where multiple sources of parental income were available on these participants (i.e. adopted father, adopted mother, father, mother incomes), we assigned participants to the first non-missing disposable family income quintile based on the aforementioned parental ordering. Participants who were missing family disposable income status (N=25 250 (1·65%); Fig. 1) were excluded from the complete case analysis (see "Missing data" in Methods and Result).

At the SAMS-level, we also controlled for population density (NAPD only) and deprivation quintiles in each year of follow-up as time-varying covariates, based on a previous methodology.[11] Briefly, population density was calculated as people per square kilometre, derived from the total population recorded in each SAMS each year in the RTP. Deprivation in each SAMS was estimated from four indicators held in the LISA register, regarding the proportion of people in each SAMS who were:

unemployed, held a criminal conviction, received social welfare, or whose income was below the national median in each year. Each deprivation indicator was z-standardised and summed with higher scores indicating SAMS with greater deprivation. We coded deprivation and population density into quintiles relative to all SAMS in Sweden in a given year.

Statistical analyses: further details

We constructed a period-stratified dataset to permit multilevel (random intercepts) proportional hazards survival analysis to account for both the nested nature of the dataset (individuals nested within SAMS) and time-varying covariates [TVC] (age group, social capital exposures, deprivation and population density) as participants aged and moved between different neighbourhoods over the follow-up period. Thus, each participant's record was stratified into periods (years) for each partial or total year of follow-up until cohort exit. We set date of entry into each period as 1 January, except for the first period of follow-up, which was either 1 January 2002 (for people aged over 14 years old and already resident in the Stockholm County catchment on this date), their 14th birthday (for people who turned 14 years old after 1 January 2002 and resident in the Stockholm Count catchment on this date) or earliest date of residency in the Stockholm County catchment (for people aged over 14 years old who moved into the Stockholm County catchment for the first time after 1 January 2002). Date of exit from each period was 31 December of that year, unless there was evidence of emigration from Sweden, change in residence to a SAMS outside of Stockholm County, date of first diagnosis of SMI, or death, whichever came first. Participants were censored from further follow-up on that date, with uncensored participants followed until 31 December 2016. Where there was evidence of a move into Stockholm County (for date of entry into follow-up) from elsewhere in Sweden, or of a move out of Stockholm County to elsewhere in Sweden in any period (for date of exit from follow-up), we randomly assigned these participants entry/exit dates (day/month) for the move in this period (year), since the RTP does not record the precise date on which a change in residence occurs, only the SAMS in which a participant is registered on 31 December each year.

Statistical analyses: sensitivity analyses

We performed four sensitivity analyses to examine the effect of possible biases on our results. First, we regenerated SAMS-level social capital scores excluding ratings from SPHC respondents (N=280; 1·2%) in 2002 who were subsequently diagnosed with SMI, to exclude the possibility that reverse causation may have explained any observed associations.

Second, to examine the extent to which information bias might have influenced our findings when SAMS-level social capital scores were based on a small number of SPHC respondents, we re-ran our

main models excluding cohort participants who had lived in any SAMS during follow-up where average social capital scores were based on fewer than five SPHC respondents. This excluded 106 SAMS (13·1%) and 67 005 participants (4·6%) from the sensitivity analysis.

Third, in *post hoc* analyses, we considered the possible issue of endogeneity in our social capital measures,[12] technically defined as correlation between our exposure and error term in the model that could be driven by unobserved confounding biases and/or selection effects that led people to live in areas with higher or lower levels of perceived social capital, including socioeconomic (dis)advantage, degree of urbanisation and genetic susceptibility to mental health problems not already captured via our control for these confounders. Under certain assumptions, an instrumental variable (IV) approach can remove endogeneity from the analysis and recover the true causal effect of the exposure on the outcome. The researcher must first identify a suitable instrumental variable that is: (i) causally associated with the exposure (i.e. social capital) (the *relevance* criterion); (ii) only associated with the outcome (incidence of mental health disorders) via the exposure (the *exclusion-restriction* criterion), and; (iii) does not share a common cause with the outcome (the *exchangeability* criterion). In practice it may be hard to identify a suitable IV. Here, we considered voter turnout percentage in each SAMS neighbourhood in the 2002 Swedish County Council elections, as reported in the 2002 SPHC survey, as an IV for social capital. We posited that neighbourhood level voter turnout in local elections should reflect local levels of social participation, ties and investment in the "associational life" of the community (criterion I - relevance)[13]; would not itself be a direct cause of the incidence of mental disorders (the proportion of people voting in your neighbourhood could not cause psychiatric disorders) (criterion ii – exclusion-restriction), and; provided we controlled for potential common causes of neighbourhood-level voter turnout and incidence of mental disorders, most importantly socioeconomic deprivation[13] and population density[14] (although not migrant density[15]), this would also meet the criterion for conditional exchangeability (criterion iii – conditional exchangeability).[16]

We considered this issue as follows: first, we re-ran polychoric factor analysis as described above, but omitting voter turnout as an item. We obtained a near-identical three-factor solution to the main results and derived SAMS-level factor scores for *political*, *welfare* and *personal* trust as before. Second, we estimated the SAMS-level correlation between these exposures and 2002 voter turnout in county council elections to assess the strength of this variable as an instrument for social capital. Given potential instability in estimates of both social capital and voter turnout in neighbourhoods in the full SPHC sample, we also reported correlations in SAMS with at least 5 SPHC respondents. Third, we adopted two instrumental variable approaches to re-analyse our survival data,[17] using a two stage

least squares regression (2SLS) and control function approach, respectively. Under the 2SLS approach, we first fitted an ordinary least squares (OLS) regression of voter turnout on social capital (here, *personal trust*). We estimated the F-statistic to determine the strength of the instrument (criterion I – relevance), where an F-statistic greater than 10 indicates a sufficiently strong instrument.[18] Next, we obtain the fitted (predicted) value of each social capital exposure from the models, which in a second stage is then substituted for our original social capital exposure(s) in our survival models. Under the control function approach, we retain the residual error term from the first OLS stage above, and in a second stage control for this in our survival models alongside our original exposure variables. Under either the 2SLS or control function approach, we entered SAMS-level deprivation and population density in 2002 as covariates to aid conditional exchangeability. We compared the results from both models alongside our main results to examine the extent to which endogeneity may have driven our main findings. Given potential instability of IV and exposure estimates in SAMS neighbourhoods with small numbers of SPHC participants, we also re-ran these IV analyses in a subsample of the dataset with SAMS with at least 5 SPHC respondents. Given initial evidence in our main models that only personal trust was associated with our mental health outcomes, and given weak correlation between voter turnout and either *political* (ρ=0.06) or *welfare* trust (ρ=0.12) (Supplementary Table 7), all IV analyses were restricted to voter turnout at county council elections as an IV for *personal* trust. SAMs level *personal trust* was statistically significantly associated with voter turnout (ρ=0.21; $p<0.05$), with an F-statistic of 27.1, indicating the latter was a strong potential instrument for SAMS-level *personal trust*.

Fourth, we inspected possible departure from proportional hazards in our main models. Unlike Cox proportional hazards models, the parametric survival models do not strictly require hazards to be proportional over the follow-up period. Nonetheless, our multilevel parametric survival model with a Weibull distribution implies proportional hazards, and so we tested for possible departure from this in our main multivariable models. Since neighbourhood-level *personal trust* emerged as the only social capital measure that showed an association with our outcomes, we limited tests to this variable. To test for departure from proportional hazards we compared our final multivariable model for each outcome (as reported in Table 3) against a model which included an additional interaction term between follow-up time and neighbourhood-level *personal trust*. We performed a likelihood ratio test (LRT) to determine evidence of non-proportional hazards, with an LRT $p<0.05$ consistent with such departure. Where any departure from proportional hazards was observed, we reported hazard ratios at one, five, ten and fifteen years, and plotted hazard ratios graphically with respect to follow-up time.

Supplementary Results

SPHC survey respondent representativeness: further details

In univariable analyses (Extended Data Table 2), there were differences between SPHC survey respondents and the remainder of the total population in the Stockholm County catchment by population density, deprivation and median values of social capital. Following multivariable modelling, however, fewer of these trends persisted. For example, SPHC respondents were representative of the total population of Stockholm in the lowest and highest quintiles of population density, although were more likely to live in areas with intermediate quintiles of population density than the general population. Differences by deprivation were inconsistent, although SPHC respondents were less likely to live in the middle and most deprived quintiles than the general population (Extended Data Table 2). SPHC respondents and the general population also shared similar levels of exposure to *political* (OR: 0·96; 95%CI: 0·84-1·10) and *personal trust* (OR: 0·96; 95%CI: 0·84-1·10), but – on average – SPHC respondents lived in areas with lower levels of *welfare trust* (OR: 0·87; 95%CI: 0·77-0·97) than the general population. Together, these results suggested that SPHC respondents differed most strongly from the general population in the Stockholm County catchment area in terms of individual level factors, with older, female, higher income, and Swedish-born groups over-represented in the SPHC survey. As detailed in the main results section, participants from Sub-Saharan Africa (OR: 0·44; 95%CI: 0·37-0·53), North Africa and the Middle East (OR: 0·59; 95%CI: 0·54-0·64) or Asia (OR: 0·59; 95%CI: 0·52-0·68) were substantively under-represented in the SPHC.

Sample characteristics: further details

Given that exposure to *personal trust* was associated with lower rates of NAPD in participants of mixed parental regions-of-origin (see Table 4), a more detailed description of parental regions-of-origin is helpful to better understand this group's recent migration history (Extended Data Table 8). In our complete case cohort, 107 422 participants were born in Sweden to biological parents who came from different regions-of-origin. Of this group, 86·37% of participants had one Swedish-born parent and one parent born elsewhere; the majority of these participants (N=55 815; 51·96% of the mixed group, and 60·16% of those with one Swedish-born parent) had one parent born in Sweden and one born in another European country (including Russia). Of those participants with two foreign-born parents from different regions-of-origin (N=14 638; 13·63% of this group), nearly all were participants whose parents were born in two different (non-Swedish) European countries (N=13 059; 12·16% of the mixed group, and 89·21% of those with two parents from different regions-of-origin). Therefore, in total, 98·53% of the mixed group (i.e. 86·37% + 12·16%) had one parent born in Sweden or two parents from different European regions-of-origin.

Geographic variance in incidence and SAMS-level correlation: further details

Incidence rates varied between SAMS neighbourhoods across Stockholm County (Fig. 2B-D; Supplementary Fig. 2). For example, the median SAMS-level crude incidence of non-affective psychotic disorders was 36·16 per 100 000 person-years (interquartile range [IQR]: 19·30-62·46).

The presence of statistically-significant SAMS-level variance in incidence of all outcomes was confirmed in null random intercepts models (Supplementary Table 4), being greatest for non-affective psychotic disorders (σ^2=0·34; 95%CI: 0·29-0·40), followed by bipolar disorder without psychosis (σ^2=0·16; 95%CI: 0·13-0·19) and affective psychotic disorders (σ^2=0·09; 95%CI: 0·05-0·14). Subsequent multilevel proportional hazards modelling demonstrated that substantial between-neighbourhood variance in incidence remained for all three outcomes following adjustment for included individual-level confounders (Supplementary Table 4). Following full multivariable modelling (see below), this variance was reduced by over 93% for non-affective and affective psychotic disorders and by over 62% for bipolar disorder without psychosis (Supplementary Table 4), suggesting inclusion of neighbourhood-level fixed effects (exposures and covariates) largely accounted for residual between-neighbourhood variance in incidence.

Correlations between SAMS-level variables were generally statistically significant, but modest (Table 2); greater *personal trust* was negatively correlated with lower deprivation (ρ=-0·22; p<0·05) and population density (ρ=-0·14; p<0·05), which were positively correlated with each other (ρ=0·23; p<0·05). *Political* and *welfare trust* were unrelated to deprivation or population density. *Welfare trust* was weakly positively correlated with *political trust* (ρ=0·12; p<0·05) and *personal trust* (ρ=0·13; p<0·05). The modest values of these correlations was born out by visual inspection of the spatial distribution of these characteristics across SAMS in Stockholm County (Extended Data Fig. 6).

Instrumental variable results: further details

Our IV approaches (2 stage least squares regression [2SLS] and control function [CF]) continued to support several findings from our main analyses (Extended Data Table 6), including an overall protective effect of neighbourhood-level *personal trust* on the incidence of non-affective psychotic disorders (HR_{2SLS}: 0·95; 95%CI: 0·91-0·99), and a similar trend for bipolar disorder without psychosis (HR_{2SLS}: 0·96; 95%CI: 0·92-1·01). Consistent with our main results, a protective effect of neighbourhood-level personal trust was evident for the Swedish-born population for both non-affective psychotic disorders and non-psychotic bipolar disorder under both instrumental variable approaches, and as per our main results, these protective effects continued to be observed for people of other European and mixed (mostly Scandinavian) countries of origin (Extended Data Table 6). By

contrast, our instrumental variable modelling found no evidence of a protective effect of neighbourhood *personal trust* on any outcome for people from other migrant backgrounds. Although effect sizes were reduced under our instrumental variable approaches compared with the main results, there was some evidence of an adverse association between higher levels of neighbourhood personal trust and the incidence of severe mental illnesses (Extended Data Table 6). For example, higher neighbourhood personal trust was associated with a greater incidence of non-affective psychotic disorders (HR_{2SLS}: 1·29; 95%CI: 1·05-1·57) and non-psychotic bipolar disorder (HR_{CF}: 3·75; 95%CI: 1·17-12·04) in people from Sub-Saharan Africa, and for non-psychotic bipolar disorder for people of North African and Middle Eastern origin (HR_{2SLS}: 1·42; 95%CI: 1·17-1·73). We also found partial novel evidence of a similar pattern for people from Asian countries (HR_{2SLS}: 1·79; 95%CI: 1·14-2·82) in one instrumental variable approach.

We noted some inconsistencies in our results for migrant groups between instrumental variable approaches, which may arise due to different identifying assumptions and functional forms of the two approaches when applied to nonlinear models.[19] Further research is required to explore this statistical issue, but we suggest caution in interpreting our instrumental variable results where findings differ between approaches; thus, the most consistent and conservative conclusion from our results is that a protective effect of neighbourhood *personal trust* existed for people of Swedish and other European origins, but that no such effect was apparent for migrants from other countries of origin.

Supplementary Figures

Supplementary Fig. 1: Screeplot following polychoric factor analysis of imputed SPHC survey data on 14 items related to social capital in Stockholm County in 2002

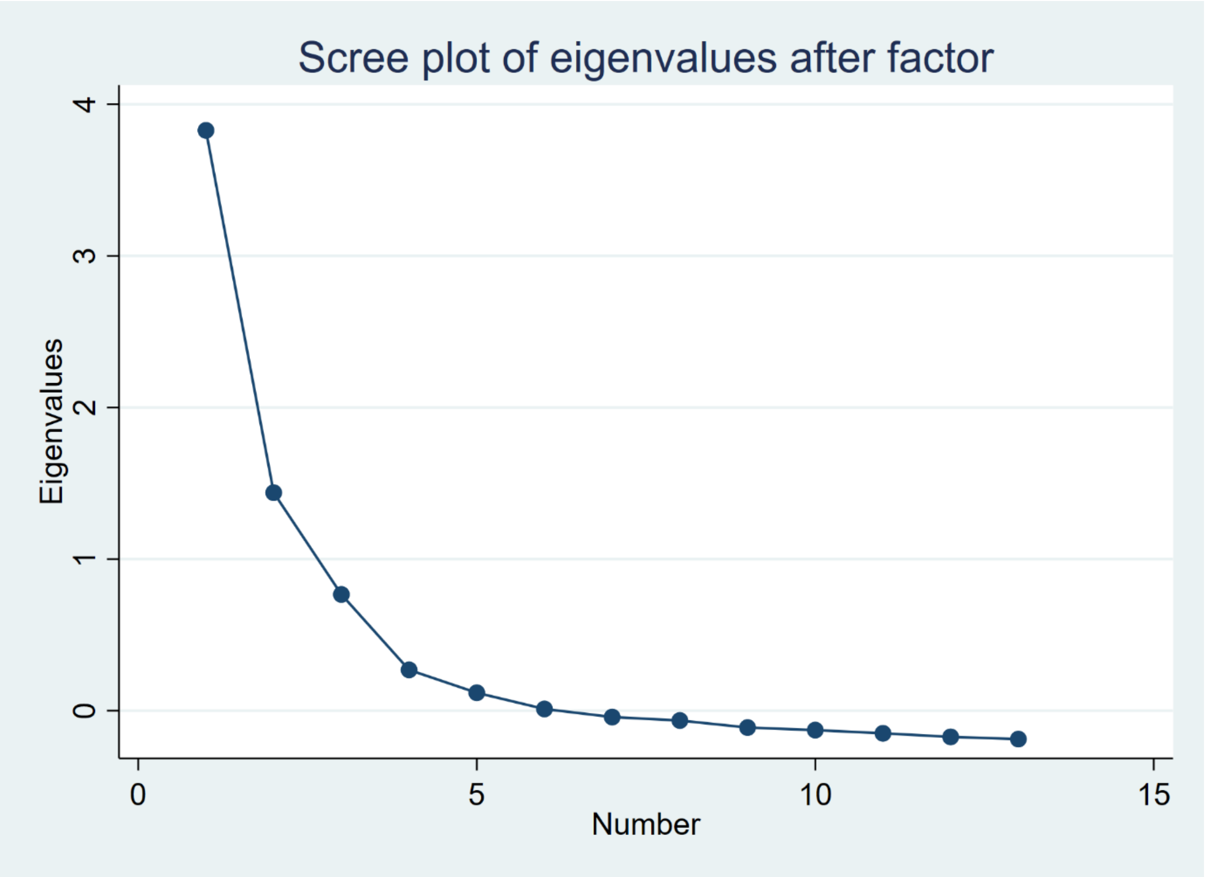

Legend: Following polychoric factor analysis of 13 included social capital items in the SPHC survey in 2002, we identified a 3-factor solution as providing optimal fit to the data, based on inspection of the screeplot and factor loadings (Extended Data Table 3).

Supplementary Fig. 2: Distribution of incidence per 100,000 person-years of each outcome at SAMS level in Stockholm County, 2002-2016 (N=820 SAMS)

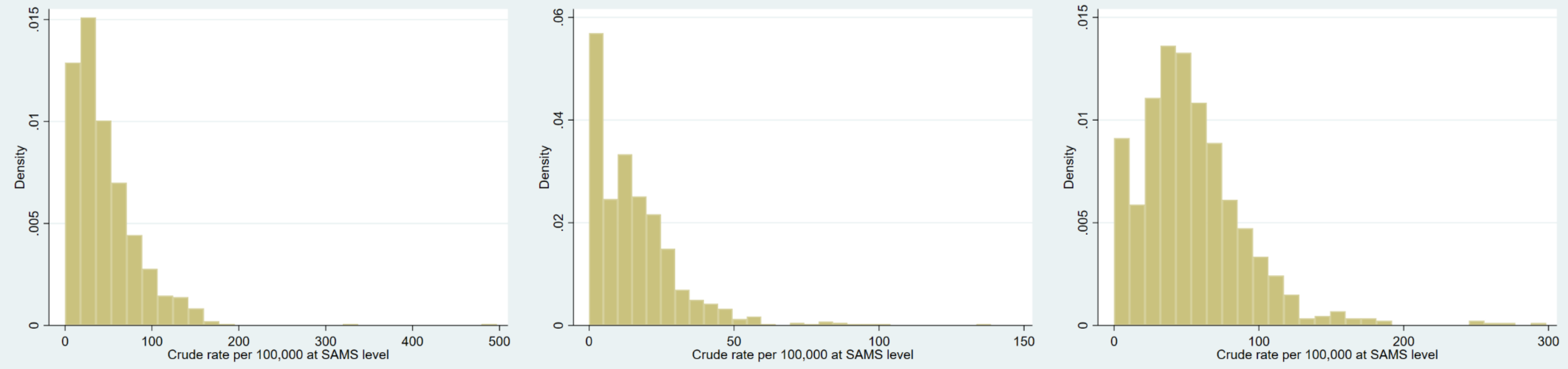

A. Non-affective psychotic disorders **B. Affective psychotic disorders** **C. Bipolar disorder without psychosis**

Supplementary Fig. 3: Directed Acyclic Graphs of the hypothesised relationship between SAMS-level social capital, confounders and non-affective psychotic disorders

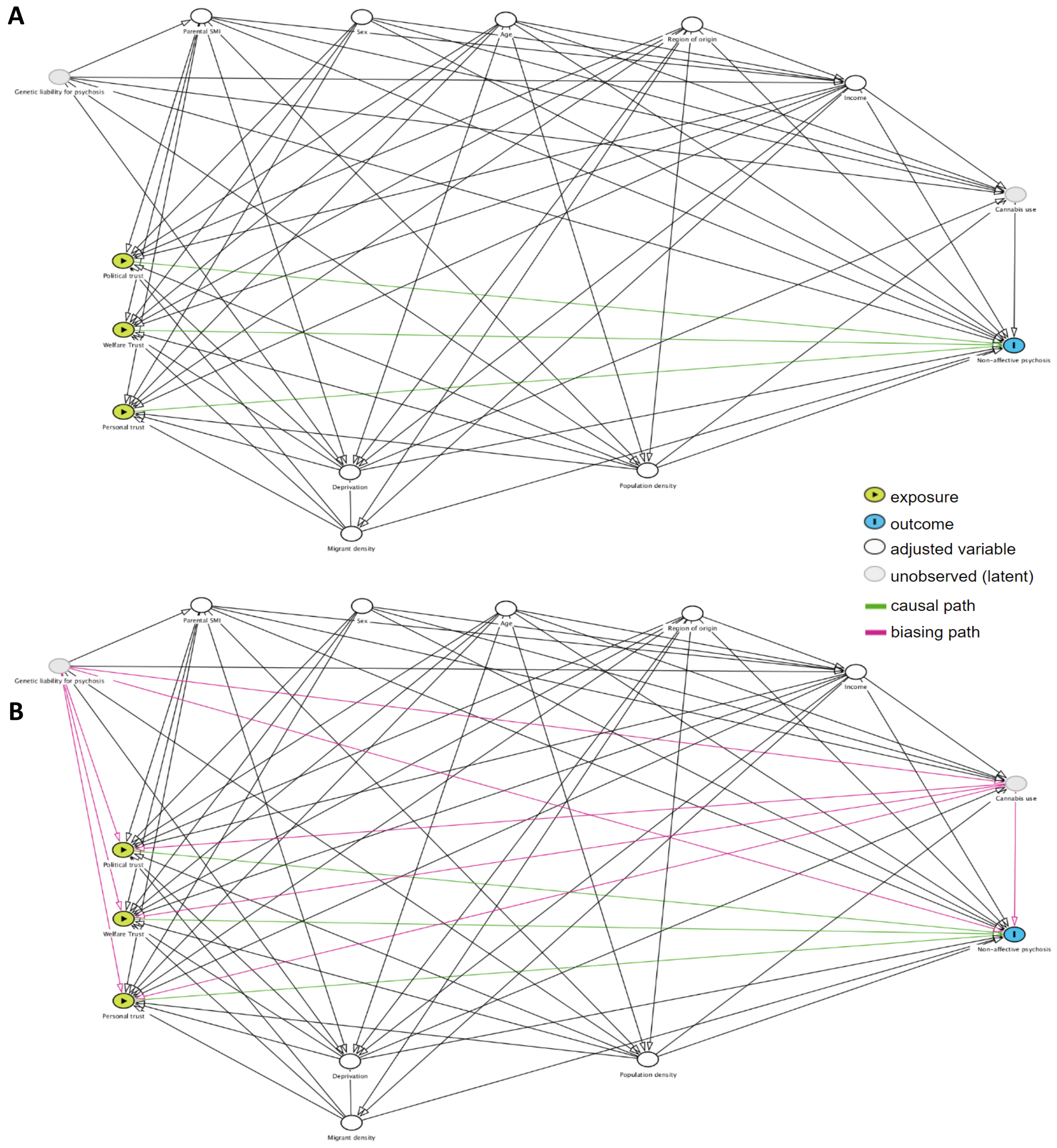

under different causal assumptions

Legend: We are interested in estimating the total causal effects (green arrows) of our three social capital exposures (*political*, *welfare* and *personal trust*; green ovals) on the incidence of non-affective psychotic disorders (blue ovals), after adjustment for a minimal set of confounding factors. The assumed causal direction of association between all variables, including unobserved (grey ovals) and observed/adjusted covariates (white ovals) is denoted by the direction of the paths. The absence of a path makes the strong assumption of no association between two variables. **Panel A** specifies one plausible causal structure underlying the problem of interest. Here, we assume that parental SMI, age, sex, parental region-of-origin, disposable income, and SAMS-level deprivation, population density and migrant density are ancestors of social capital (our exposures) and non-affective psychotic disorders (outcome), based on extensive prior evidence (see main manuscript for references). Other causal relationships are also depicted in the DAG. We make the strong assumption there is no causal effect between individual-level cannabis use and area-level social capital. This assumption may be valid in the presence of a low prevalence of cannabis use in the population, as appears to be the case in Sweden, where the 1-month prevalence of cannabis use is estimated to be around 1%.[20] We also make the strong assumption that any causal effect of genes for SMI on area-level social capital are mediated via our observed variable of parental history of SMI. Adjustment for this observed covariate structure closes (i.e. blocks) all biasing paths (black arrows) to allow estimation of the causal effect of social capital on non-affective psychotic disorders. If either of these assumptions are invalid, unobserved confounding

due to genes for SMI and/or cannabis would not allow us to estimate the total causal effect of social capital on non-affective psychotic disorders (**Panel B**).

Supplementary Fig. 4: Directed Acyclic Graphs of the hypothesised relationship between SAMS-level social capital, confounders and affective psychotic disorders or non-psychotic bipolar disorder under different causal assumptions

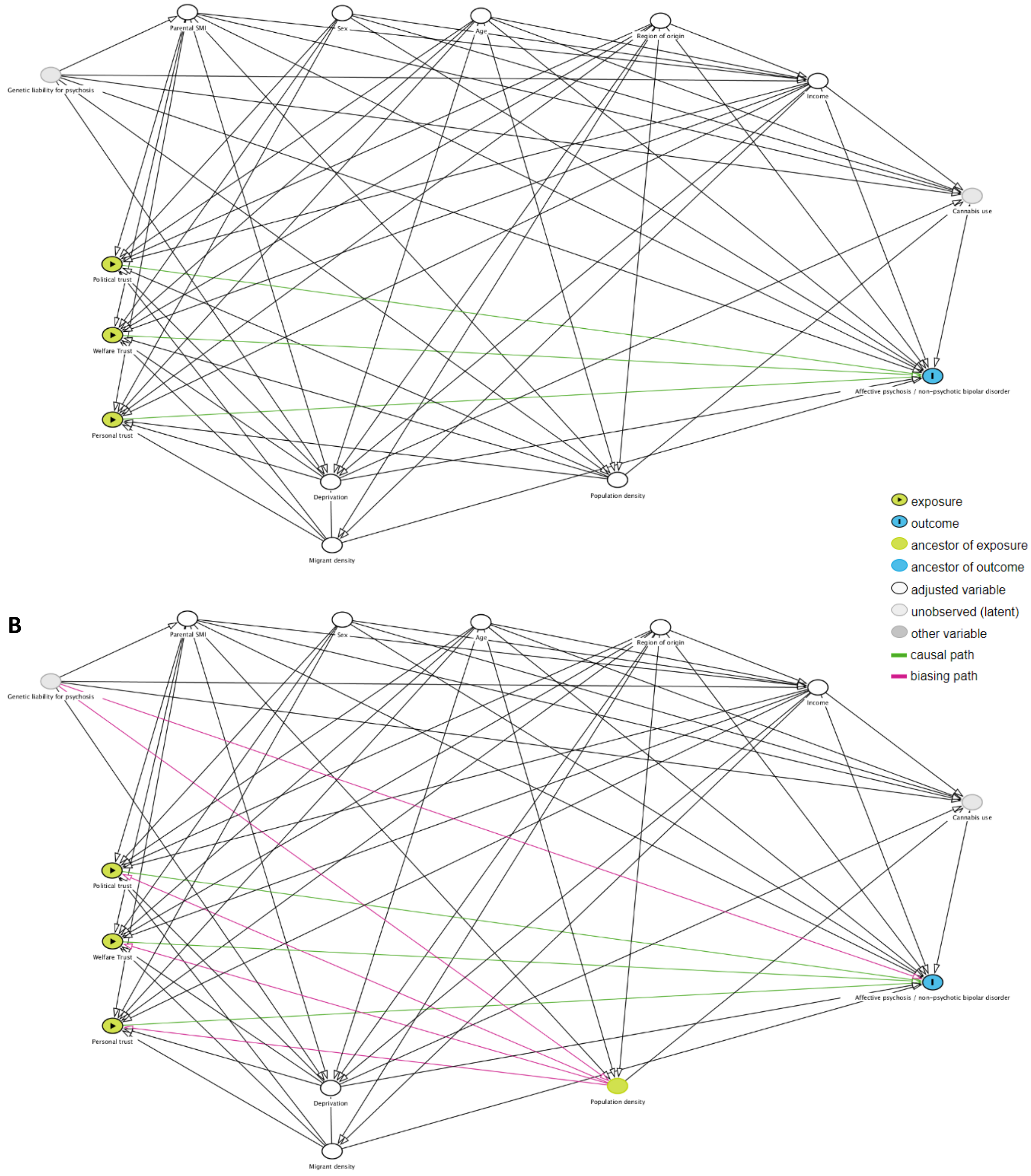

Legend: Panel A shows the same hypothesised causal structure underlying the relationship between social capital and affective psychotic disorders or bipolar disorder without psychosis (which we assume share the same pattern) as described for non-affective psychoses in Supplementary Fig. 3A, except we assume no direct causal association between population density, cannabis use and these outcomes, consistent with current evidence.[21,22] Nonetheless, the same assumptions underlie this model as described above. Further, although no direct relationship is assumed to exist between population density and these outcomes, it remains necessary to condition on this variable in analyses, given the biasing path otherwise induced via failure to block the indirect association via unobserved genes for SMI (**Panel B**). We also note that no such biasing path is introduced by the failure to adjust for cannabis use in this DAG. Thus, the minimal adjustment set for all outcomes in our study are identical: parental SMI, sex, age, region-of-origin, income, deprivation, population density and migrant density.

Supplementary Fig. 5: Flowchart of respondents to the 2002 Stockholm Public Health Cohort social capital items

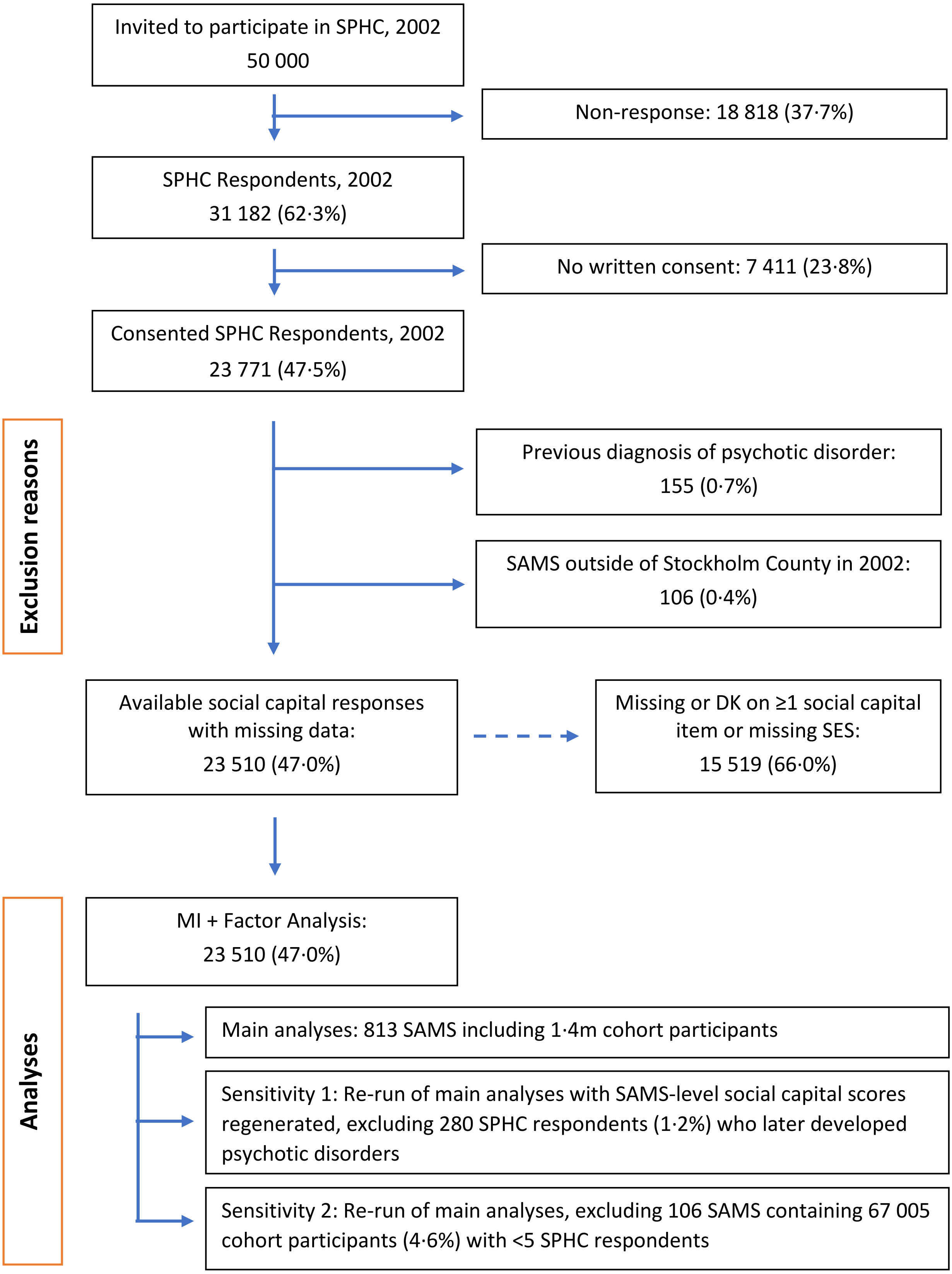

Supplementary Tables

Supplementary Table 1: Sample characteristics by missing data status

	Complete case sample		Missing data		Association	
	N	%	N	%	χ^2 (df)	*p*
**Total**	1 467 128	96.07	60 151	3·93		
**Sex**					298·3 (1)	<0·01
Male	736 039	95·79	32 338	4·21		
Female	731 089	96·34	27 813	3·66		
**Age group (cohort exit)**					13 158·6 (9)	<0·01
14-19	130 963	91·23	12 585	8·77		
20-24	170 411	95·97	7 163	4·03		
25-29	157 433	94·37	9 399	5·63		
30-34	128 665	96·33	4 896	3·67		
35-39	116 394	96·55	4 154	3·45		
40-44	119 007	97·06	3 602	2·94		
45-49	115 867	97·46	3 014	2·54		
50-54	116 807	97·64	2 822	2·36		
55-59	99 149	97·61	2 425	3·13		
60-64	312 432	96·87	10 091	3·94		
**Migrant status**					1 164·6 (1)	<0·01
Swedish-born	1 181 774	96·75	39 707	3·25		
Children of migrants	285 354	95·47	13 543	4·53		
Missing	-	-	6 901	-		
**Region-of-origin**					2 357·0 (6)	<0·01
Sweden	1 181 774	96·75	39 707	3·25		
Other Europe	124 606	96·45	4 589	3·55		
Asia	5 507	92·65	437	7·35		
North Africa & Middle East	33 399	92·98	2 521	7·02		
Sub-Saharan Africa	8 277	94·61	472	5·39		
Mixed	107 422	95·39	5 191	4·61		
Other	6 143	94·86	333	5·14		
Missing	-	-	6 901	-		
**Parental history of SMI**					30·5 (1)	<0·01
No	1 407 496	96·04	57 961	3·96		
Yes	59 632	96·49	2 172	3·51		
**Family disposable income (cohort entry)[a]**					3 006·5	<0.01
1 – Lowest	231 125	96·36	8 724	3·64		
2	246 789	97·43	6 511	2·57		
3	255 561	97·74	5 909	2·26		
4	246 270	98·08	4 815	1·92		
5 – Highest	487 383	98·31	8 356	1·69		
Missing	-	-	25 250	-		
**Population density (cohort entry)[a]**					217·3 (4)	<0·01
1 – Lowest	9 281	98·27	163	1·73		
2	64 490	98·04	1 287	1·96		
3	91 953	98·16	1 720	1·84		
4	425 157	98·04	8 482	1·96		
5 – Highest	876 247	97·72	20 475	2·28		
Missing	-	-	28 000	-		
**Deprivation (cohort entry)[a]**					595·9 (4)	<0·01
1 – Lowest	480 659	97·75	11 062	2·25		
2	345 664	97·46	9 007	2·54		
3	288 069	98·26	5 099	1·74		
4	171 576	98·04	3 428	1·96		
5 – Highest	181 160	98·09	3 531	1·91		

Missing	-	-	28 000	-		
**SMI**					22·1 (1)	<0·01
No	1 449 368	96·05	59 551	3·95		
Yes	17 760	96·73	600	3·27		
**Social capital (cohort entry)**	*Median*	*IQR*	*Median*	*IQR*		
Political trust	0·03	-0·05 – 0·10	0·03	-0·05 – 0·09	-3·2	<0·01
Welfare trust	-0·02	-0·16 – 0·11	0·00	-0·15 – 0·11	4·6	<0·01
Personal trust	0·06	-0·26 – 0·44	0·09	-0·25 – 0·46	5·2	<0·01

IQR: interquartile range; df: degrees of freedom; SMI: severe mental illness

[a] Relative to whole of Sweden

Supplementary Table 2: Complete case sample characteristics by affective psychosis diagnosis status

	Population at-risk[a]		Affective psychosis		Association	
	**N**	**%**	**N**	**%**	**χ² (df)**	***p***
**Total**	1 464 771	99·84	2 357	0·16		
**Sex**					24·3 (1)	<0.001
Male	734 976	99·86	1 063	0·14		
Female	729 795	99·82	1 294	0·18		
**Age group (cohort exit)**					279.6 (9)	<0·001
14-19	130 790	99·87	173	0·13		
20-24	170 167	99·86	244	0·14		
25-29	157 170	99·83	263	0·16		
30-34	128 435	99·82	230	0·18		
35-39	116 157	99·80	237	0·20		
40-44	118 764	99·80	243	0·20		
45-49	115 628	99·79	239	0·21		
50-54	116 551	99·78	256	0·22		
55-59	98 903	99·75	246	0·25		
60-64	312 206	99·93	226	0·07		
**Migrant status**					29·1 (1)	<0·001
Swedish-born	1 179 979	99·85	1 795	0·15		
Children of migrants	284 792	99·80	562	0·20		
**Region-of-origin**					44·0 (6)	<0·001
Sweden	1 179 979	99·85	1 795	0·15		
Other Europe	124 344	99·79	262	0·21		
Asia	5 503	>99·90	<5[b]	<0·10[b]		
North Africa & Middle East	33 349	99·85	50	0·15		
Sub-Saharan Africa	8 264	99·84	13	0·16		
Mixed	107 197	99·79	225	0·21		
Other	6 135	99·87	<10[b]	<0·15[b]		
**Parental history of SMI**					304·7 (1)	<0·001
No	1 405 402	99·85	2 094	0·15		
Yes	59 369	98·56	263	0·44		
**Family disposable income (cohort entry)[c]**					92.4 (4)	<0.001
1 – Lowest	230 677	99·81	448	0·19		
2	246 278	99·79	511	0·21		
3	255 156	99·84	405	0·16		
4	245 876	99·84	394	0·16		
5 – Highest	486 784	99·88	599	0·12		
**Population density (cohort entry)[c]**					25·9 (4)	<0·001
1 – Lowest	9 268	99·86	13	0·14		
2	64 395	99·85	95	0·15		
3	91 833	99·87	120	0·13		
4	424 555	99·86	602	0·14		
5 – Highest	874 720	99·83	1 527	0·17		
**Deprivation (cohort entry)[c]**					93·0 (4)	<0·001
1 – Lowest	480 024	99·87	635	0·13		
2	345 187	99·86	477	0·14		
3	287 556	99·82	513	0·18		
4	171 243	99·81	333	0·19		
5 – Highest	180 761	99·78	399	0·22		
**Social capital (cohort entry)**	*Median*	*IQR*	*Median*	*IQR*		
Political trust	0·04	-0·06 – 0·10	0·04	-0·05 – 0·11	-1·2	0·25
Welfare trust	-0·03	-0·15 – 0·10	-0·03	-0·16 – 0·09	1·8	0·07
Personal trust	0·08	-0·27 – 0·44	-0·03	-0·34 – 0·39	6·0	<0·0001

df: degrees of freedom; IQR: Interquartile range

[a] Remainder of the complete case sample

[b] Values suppressed due to possible risk of disclosure in cells where n<5

[c] Relative to whole of Sweden

Supplementary Table 3: Complete case sample characteristics by bipolar disorder without psychosis diagnosis status

	Population at-risk[a]		Bipolar disorder w/o psychosis		Association	
	**N**	**%**	**N**	**%**	**χ² (df)**	***p***
**Total**	1 459 016	99.45	8 112	0.55		
**Sex**					623·9 (1)	<0.001
Male	733 091	99·60	2 948	0·40		
Female	725 925	99·29	5 164	0·71		
**Age group (cohort exit)**					1 660.9 (9)	<0·001
14-19	130 349	99·53	614	0·47		
20-24	169 264	99·33	1 147	0·67		
25-29	156 343	99·31	1 090	0·69		
30-34	127 684	99·24	981	0·76		
35-39	115 408	99·15	986	0·85		
40-44	118 089	99·23	918	0·77		
45-49	115 044	99·29	823	0·71		
50-54	116 124	99·42	683	0·58		
55-59	98 633	99·48	516	0·52		
60-64	312 078	98·89	354	0·11		
**Migrant status**					17·4 (1)	<0·001
Swedish-born	1 175 388	99·46	6 386	0·54		
Children of migrants	283 628	99·40	1 726	0·60		
**Region-of-origin**					190·2 (6)	<0·001
Sweden	1 175 388	99·46	6 386	0·54		
Other Europe	123 710	99·28	896	0·72		
Asia	5 493	99·75	14	0·25		
North Africa & Middle East	33 324	99·78	75	0·22		
Sub-Saharan Africa	8 266	99·87	11	0·13		
Mixed	106 717	99·34	705	0·66		
Other	6 118	99·59	25	0·41		
**Parental history of SMI**					1 063·1 (1)	<0·001
No	1 400 292	99·49	7 204	0·51		
Yes	58 724	98·48	908	1·52		
**Family disposable income (cohort entry)[b]**					651·7 (4)	<0·001
1 – Lowest	229 211	99·17	1 914	0·83		
2	245 116	99·32	1 673	0·68		
3	254 180	99·46	1 381	0·54		
4	244 990	99·48	1 280	0·52		
5 – Highest	485 519	99·62	1 864	0·38		
**Population density (cohort entry)[b]**					50·4 (4)	<0·001
1 – Lowest	9 226	99·41	55	0·59		
2	64 114	99·42	376	0·58		
3	91 468	99·47	485	0·53		
4	423 081	99·51	2 076	0·49		
5 – Highest	871 127	99·42	5 120	0·58		
**Deprivation (cohort entry)[b]**					232·6 (4)	<0·001
1 – Lowest	478 530	99·56	2 129	0·44		
2	343 820	99·47	1 844	0·53		
3	286 332	99·40	1 737	0·60		
4	170 353	99·29	1 223	0·71		
5 – Highest	179 981	99·35	1 179	0·65		
**Social capital (cohort entry)**	*Median*	*IQR*	*Median*	*IQR*		
Political trust	0·04	-0·06 – 0·10	0·04	-0·05 – 0·11	-3·2	0·001
Welfare trust	-0·03	-0·16 – 0·10	-0·03	-0·16 – 0·10	2·0	0·048
Personal trust	0·08	-0·27 – 0·44	-0·01	-0·31 – 0·39	7·4	<0·0001

df: degrees of freedom; IQR: Interquartile range

[a] Remainder of the complete case sample

[b] Relative to whole of Sweden

Supplementary Table 4: Variance partition coefficients from multilevel modelling of psychiatric outcomes

	Residual variance		% SAMS variance explained	Statistical significance
**Model adjustment**	**σ^2**	**95% CI**		**χ^2 (df); p-value**
**Non-Affective psychotic disorders**				
**Null model**	0·34	0·29-0·40	-	142·3 (1); <0·0001
**Individual-level variables**[a]	0·15	0·13-0·20	54·4%	78·9 (1); <0·0001
**Full multivariable**[b]	0·02	0·01-0·04	93·0%	12·3 (1); 0·0005
**Full multivariable with interaction**[c]	0·02	0·01-0·04	92·9%	12·7 (1); 0·0004
**Affective psychotic disorders**				
**Null model**	0·09	0·05-0·14	-	15·6 (1); 0·0001
**Individual-level variables**[a]	0·04	0·02-0·10	52·6%	5·4 (1); 0·02
**Full multivariable**[b]	0·00	0·00-1·50	95·0%	0·1 (1); 0·74
**Bipolar disorder without psychosis**				
**Null model**	0·16	0·13-0·19	-	107·4 (1); <0·0001
**Individual-level variables**[a]	0·10	0·08-0·13	36·9%	74·4 (1); <0·0001
**Full multivariable**[b]	0·06	0·04-0·08	62·7%	42·8 (1); <0·0001
**Full multivariable with interaction**[c]	0·06	0·04-0·08	63·7%	41·6 (1); <0·0001

σ^2: sigma squared, or residual between-SAMS variance in incidence after model fit; CI: confidence interval; df: degrees of freedom;

[a] Multivariable multilevel survival model adjust for individual-level covariates only: age group, sex, their interaction, parental history of SMI, region-of-origin, family disposable income quintile at cohort entry.

[b] Final multivariable multilevel survival model, adjusted for age group, sex, their interaction, parental history of SMI, parental region-of-origin, family disposable income quintile at cohort entry, and time-varying deprivation quintile, own-group migrant density, and other social capital domains. For non-affective psychotic disorders, we additionally adjusted for time-varying population density quintile. See Supplementary Fig. 3 and 4.

[c] Full multivariable model, including interaction between region-of-origin and personal trust (Tables 3 and 4).

Supplementary Table 5: Association between item-level missingness and sociodemographic characteristics for social capital measures from the Stockholm County Public Health Cohort in 2002

**Item**	**Item-level missingness** **N (%)**[a]	**Age group** **Z; p-value**[b]	**Sex** **X2 (df); p-value**	**Region-of-origin** **X^2 (df); p-value**	**Relationship status** **X^2 (df); p-value**	**Socioeconomic status** **X^2 (df); p-value**	**Deprivation quintile** **X^2 (df); p-value**	**Population density quintile** **X^2 (df); p-value**
Trust in healthcare	780 (3·3)	10·3; <0·001	5·1 (1); 0·02	118·3 (1); <0·001	33·3 (2); <0·001	84·1 (5); <0·001	54·9 (4); <0·001	4·9 (4); 0·30
Trust in social services	10 426 (44·4)	25·0; <0·001	34·7 (1); <0·001	10·7 (1); 0·001	177·3 (2); <0·001	86·2 (5); <0·001	44·5 (4); <0·001	14·3 (4); 0·007
Trust in insurance fund	6 657 (28·3)	26·8; <0·001	4·2 (1); 0·04	21·1 (1); <0·001	37·1 (2); <0·001	53·0 (5); <0·001	15·7 (4); 0·003	8·0 (4); 0·09
Trust in employment services	10 607 (45·1)	52·8; <0·001	43·2 (1); <0·001	5·2 (1); 0·02	896·9 (2); <0·001	154·6 (5); <0·001	61·2 (4); <0·001	19·9 (4); 0·001
Trust in police	2 358 (10·0)	26·4; <0·001	191·5 (1); <0·001	208·2 (1); <0·001	262·3 (2); <0·001	278·9 (5); <0·001	91·4 (4); <0·001	4·3 (4); 0·37
Trust in parliament	3 214 (13·4)	14·3; <0·001	323·9 (1); <0·001	272·8 (1); <0·001	106·4 (2); <0·001	598·7 (5); <0·001	153·4 (4); <0·001	16·5 (4); 0·002
Trust in government	2 863 (12·2)	10·2; <0·001	279·0 (1); <0·001	257·6 (1); <0·001	65·7 (12; <0·001	538·0 (5); <0·001	131·9 (4); <0·001	14·6 (4); 0·006
Trust in county council politicians	5 648 (24·0)	-8·6; <0·001	207·3 (1); <0·001	52·4 (1); <0·001	108·9 (2); <0·001	229·1 (5); <0·001	63·4 (4); <0·001	26·9 (4); <0·001
Trust in municipal politicians	4 715 (20·1)	-5·3; <0·001	211·6 (1); <0·001	101·8 (1); <0·001	123·9 (2); <0·001	357·5 (5); <0·001	183·5 (4); <0·001	86·2 (4); <0·001
Support in crisis	277 (1·0)	4·5; <0·001	0·3 (1); 0·57	20·4 (1); <0·001	4·3 (2); 0·12	9·5 (5); 0·09	5·4 (4); 0·25	4·7 (4); 0·32
Help in illness	237 (1·0)	4·8; <0·001	0·1 (1); 0·81	22·5 (1); <0·001	11·2 (2); 0·004	16·5 (5); 0·005	9·9 (4); 0·04	2·6 (4); 0·62
Trust in residential area	527 (2·2)	8·6; <0·001	7·5 (1); 0·006	57·6 (1); <0·001	62·2 (1); <0·001	41·9 (5); <0·001	52·1 (4); <0·001	18·4 (4); 0·001
Regularly participate in activities	234 (1·0)	3·1; 0·002	3·7 (1); 0·05	21·8 (1); <0·001	10·2 (2); 0·006	7·0 (5); 0·22	3·0 (4); 0·56	2·0 (4); 0·73
Voted in any 2002 election[c]	0 (0·0)	N/A	N/A	N/A	N/A	N/A	N/A	N/A

df: degrees of freedom; N/A: not applicable; SPHC: Stockholm Public Health Cohort

[a] Of 23 510 people who consented to participate in the 2002 Stockholm Public Health Cohort survey in 2002, and who provided a response about a SAMS area in Stockholm County, with no previous history of severe mental illness in the National Patient Register (see also Supplementary Fig. 5).

[b] Mann-Whitney U-test Z-statistic to determine whether the age distribution of SPHC respondents in 2002 differed for those with complete or missing item-level responses to social capital item in the SPHC survey. Negative Z values denoted that older age was associated with missing values.

[c] No missing data

Supplementary Table 6: Cross-validation of factor structure using k-fold cross-validation (k=10) to estimate the polychoric factor structure of the social capital model

	**Factor 1: Political Trust**	**Factor 2: Welfare Trust**	**Factor 3: Personal Trust**
RMSE	0·0072	0·0057	0·0046
MAE	<0·0001	0·0001	<0·0001

RMSE; Root Mean Square Error; Mean Absolute Error

Supplementary Table 7: Correlation between median levels of SAMS-level trust variables, population density, deprivation and voter turnout for instrumental variable analyses (criterion i – relevance)[a]

	**Political trust**	**Welfare trust**	**Personal trust**	**Population density**	**Deprivation**	**Voter turnout**
	**ρ**	**ρ**	**ρ**	**ρ**	**ρ**	**ρ**
**Political trust**	1					
**Welfare trust**	0·18*	1				
**Personal trust**	-0·01	0·05	1			
**Population density**	-0·06	-0·04	-0·12*	1		
**Deprivation**	-0·07*	0·02	-0·21*	0·23*	1	
**Voter turnout**	-0·05	0·12*	0·21*	-0·06	-0·16*	1

*p<0.05

[a] In the first stage of the 2SLS instrumental variable analysis, we estimated an F-statistic of 27.1 when *personal trust* was regressed on voter turnout (conditional on population density and deprivation) at the SAMS-level, indicating voter turnout was potentially a strong instrument for *personal trust* (criterion i – relevance).

Supplementary references

1. Andersson, R. & Musterd, S. What scale matters? Exploring the relationships between individuals' social position, neighbourhod context and the scale of neighbourhood. *Source: Geografiska Annaler. Series B, Human Geography* **92**, 23–43 (2010).

2. Dykxhoorn, J. *et al.* Risk of schizophrenia, schizoaffective, and bipolar disorders by migrant status, region of origin, and age-at-migration: A national cohort study of 1.8 million people. *Psychol Med* **49**, 2354–2363 (2018).

3. Svensson, A. C. *et al.* Cohort Profile: The Stockholm Public Health Cohort. *Int J Epidemiol* **42**, 1263–1272 (2013).

4. Rubin, D. B., Stern, H. S. & Vehovar, V. Handling 'Don't Know' Survey Responses: The Case of the Slovenian Plebiscite. *J Am Stat Assoc* **90**, 822 (1995).

5. Durand, R. M. & Lambert, Z. v. Don't know responses in surveys: Analyses and interpretational consequences. *J Bus Res* **16**, 169–188 (1988).

6. Sterne, J. A. C. *et al.* Multiple imputation for missing data in epidemiological and clinical research: Potential and pitfalls. *BMJ (Online)* **339**, 157–160 (2009).

7. Madley-Dowd, P., Hughes, R., Tilling, K. & Heron, J. The proportion of missing data should not be used to guide decisions on multiple imputation. *J Clin Epidemiol* **110**, 63–73 (2019).

8. O'Brien, R. M. Estimating the Reliability of Aggregate-Level Variables Based on Individual-Level Characteristics: *Sociol Methods Res* **18**, 473–504 (2016).

9. Hawkins, D. M. The Problem of Overfitting. *J Chem Inf Comput Sci* **44**, 1–12 (2004).

10. Pedersen, C. B. & Mortensen, P. B. Urbanicity during upbringing and bipolar affective disorders in Denmark. *Bipolar Disord* **8**, 242–247 (2006).

11. Dykxhoorn, J., Lewis, G., Hollander, A. C., Kirkbride, J. B. & Dalman, C. Association of neighbourhood migrant density and risk of non-affective psychosis: a national, longitudinal cohort study. *Lancet Psychiatry* **7**, 327–336 (2020).

12. Howley, P. Addressing endogeneity in estimating the effect of social capital on psychological health. *http://dx.doi.org/10.1080/13504851.2014.927561* **22**, 76–79 (2014).

13. Islam, M. K. *et al.* Does it really matter where you live? A panel data multilevel analysis of Swedish municipality-level social capital on individual health-related quality of life. *Health Econ Policy Law* **1**, 209–235 (2006).

14. Preuss, G. G. The effects of density and urban residence on voter turnout. *Population and Environment 1981 4:4* **4**, 246–265 (1981).

15. Andersson, H., Lajevardi, N., Lindgren, K. O. & Oskarsson, S. Effects of Settlement into Ethnic Enclaves on Immigrant Voter Turnout. *Journal of Politics* **84**, 578–584 (2022).

16. Hernan MA & Robins JM. *Causal Inference: What If*. (Chapman & Hall / CRC, 2020).

17. Tchetgen, E. J. T., Walter, S., Vansteelandt, S., Martinussen, T. & Glymour, M. Instrumental variable estimation in a survival context. *Epidemiology* **26**, 402 (2015).

18. Manthey, J., Freeman, T. P., Kilian, C., López-Pelayo, H. & Rehm, J. Public health monitoring of cannabis use in Europe: prevalence of use, cannabis potency, and treatment rates. *The Lancet Regional Health - Europe* **10**, 100227 (2021).

19. Agerbo, E. *et al.* Polygenic risk score, parental socioeconomic status, family history of psychiatric disorders, and the risk for schizophrenia: A Danish population-based study and meta-analysis. *JAMA Psychiatry* **72**, 635–641 (2015).

20. Jefsen, O. H., Speed, M., Speed, D. & Østergaard, S. D. Bipolar disorder and cannabis use: A bidirectional two-sample Mendelian randomization study. *Addiction Biology* (2021) doi:10.1111/adb.13030.

21. March, D. *et al.* Psychosis and place. *Epidemiol Rev* **30**, 84–100 (2008).

22. Nahapiet, J. & Ghoshal, S. Social Capital, Intellectual Capital, and the Organizational Advantage. *Academy of Management Review* **23**, 242–266 (1998).

23. Putnam, R. *Bowling Alone: The Collapse and Revival of American Community*. (Simon and Schuster, 2000).

24. Coleman, J. S. Social Capital in the Creation of Human-Capital. *American Journal of Sociology* **94**, S95–S120 (1988).

25. Szreter, S. & Woolcock, M. Health by association? Social capital, social theory, and the political economy of public health. *Int. J. Epidemiol.* **33**, 650–667 (2004).

26. Bourdieu, P. The Forms of Capital. in *Handbook of Theory and Research for Sociology of Education* (ed. Richardson, J.) 241–258 (Greenwood Press, 1986).

27. Putnam, R. D. Bowling Alone: America's Declining Social Capital. *Journal of Democracy* **6**, 65–78 (1995).

28. Newton, K. Social Capital and Democracy: *American Behavioral Scientist* 575–586 (2016) doi:10.1177/0002764297040005004.
