# Supplementary material for: Longitudinal association between neighborhood-level social capital and incidence of major psychiatric disorders in a cohort of 1.4 million people in Sweden
Source: Nat Ment Health. 2025 Oct 20;3(11):1425–37. doi: 10.1038/s44220-025-00518-z (PMC12589101; doi:10.1038/s44220-025-00518-z)
Supplement: Supplementary file 2 — Reporting Summary [file 44220_2025_518_MOESM2_ESM.pdf]

nature portfolio

Corresponding author(s): James B. Kirkbride
Last updated by author(s): Aug 20, 2025

Reporting Summary

Nature Portfolio wishes to improve the reproducibility of the work that we publish. This form provides structure for consistency and transparency in reporting. For further information on Nature Portfolio policies, see our Editorial Policies and the Editorial Policy Checklist.

Statistics

For all statistical analyses, confirm that the following items are present in the figure legend, table legend, main text, or Methods section.

n/a	Confirmed	
☐	☒	The exact sample size (*n*) for each experimental group/condition, given as a discrete number and unit of measurement
☐	☒	A statement on whether measurements were taken from distinct samples or whether the same sample was measured repeatedly
☐	☒	The statistical test(s) used AND whether they are one- or two-sided<br>*Only common tests should be described solely by name; describe more complex techniques in the Methods section.*
☐	☒	A description of all covariates tested
☐	☒	A description of any assumptions or corrections, such as tests of normality and adjustment for multiple comparisons
☐	☒	A full description of the statistical parameters including central tendency (e.g. means) or other basic estimates (e.g. regression coefficient) AND variation (e.g. standard deviation) or associated estimates of uncertainty (e.g. confidence intervals)
☐	☒	For null hypothesis testing, the test statistic (e.g. *F*, *t*, *r*) with confidence intervals, effect sizes, degrees of freedom and *P* value noted<br>*Give P values as exact values whenever suitable.*
☒	☐	For Bayesian analysis, information on the choice of priors and Markov chain Monte Carlo settings
☐	☒	For hierarchical and complex designs, identification of the appropriate level for tests and full reporting of outcomes
☐	☒	Estimates of effect sizes (e.g. Cohen's *d*, Pearson's *r*), indicating how they were calculated

Our web collection on statistics for biologists contains articles on many of the points above.

Software and code

Policy information about availability of computer code

Data collection	No software was used.
Data analysis	Data analysis was conducted in Stata (StataCorp) version 18.2, using in-built commands and user-written code. This code is made available via our Open Sciences Framework repository, available at: https://doi.org/10.31234/osf.io/269rx

For manuscripts utilizing custom algorithms or software that are central to the research but not yet described in published literature, software must be made available to editors and reviewers. We strongly encourage code deposition in a community repository (e.g. GitHub). See the Nature Portfolio guidelines for submitting code & software for further information.

Data

Policy information about availability of data

All manuscripts must include a data availability statement. This statement should provide the following information, where applicable:

- Accession codes, unique identifiers, or web links for publicly available datasets
- A description of any restrictions on data availability
- For clinical datasets or third party data, please ensure that the statement adheres to our policy

Data for this study is available via controlled access due to ethical and legal issues surrounding the use of linked Swedish registry data and Stockholm Public Health Cohort data for research. These datasets are available via controlled access, and parties interested in using this data should contact Statistics Sweden (https://www.scb.se/en/) or the Swedish National Data Service (https://snd.gu.se/en/catalogue/study/ext0171).

Research involving human participants, their data, or biological material

Policy information about studies with human participants or human data. See also policy information about sex, gender (identity/presentation), and sexual orientation and race, ethnicity and racism.

Reporting on sex and gender	All findings presented in this study relate to sex (the biological attribute) not gender (the social construct). Findings are for all sexes combined, except where stated in the text.
Reporting on race, ethnicity, or other socially relevant groupings	Race or ethnicity are not reported in this study. All participants included in this study were Swedish-born, and we distinguinished between those born in Sweden to two Swedish born parents, versus those born in Sweden to at least one parent who was born overseas. We assigned people to groups based on their parental region(s) of origin as: Swedish-born, Other Europe, Asia, North Africa & Middle East, Sub-Saharan Africa, Mixed, and Other. These groupings allowed us to examine differences in the association between neighbourhood levels of trust and future risk of severe mental illnesses by parental region-of-origin.
Population characteristics	We included age group (14-19, then 5-year age bands until 60-64 years), history of any severe mental llness (as defined in the paper) in a biological parent, migrant status (Swedish-born to two Swedish-born parents, or children of migrants), disposable family income quintile at cohort entry, and time-varying deprivation quintile and, for non-affective psychotic disorders only, time-varying population density quintile as confounders in our analyses and to examine population characteristics of our sample.
Recruitment	Participants were identified according to our inclusion criteria from the Swedish national register data.
Ethics oversight	This study was approved by the Stockholm Regional Ethical Review Board (2010/1185-31/5) and the UCL Research Ethics Committee (21019/001).

Note that full information on the approval of the study protocol must also be provided in the manuscript.

Field-specific reporting

Please select the one below that is the best fit for your research. If you are not sure, read the appropriate sections before making your selection.

☐ Life sciences ☒ Behavioural & social sciences ☐ Ecological, evolutionary & environmental sciences

For a reference copy of the document with all sections, see nature.com/documents/nr-reporting-summary-flat.pdf

Behavioural & social sciences study design

All studies must disclose on these points even when the disclosure is negative.

Study description	Cohort study of quantitative data
Research sample	All people who were born in Sweden, and who lived in Stockholm County while aged 14-64 years old from 1 January 2002 up to 31 December 2016. Identifed from the Register of the Total Population. The sample is a complete representation of this population.Sample restricted to Stockholm County (vis-a-vis) all of Sweden, as we only had data on our exposure (neighbourhood levels of trust) from the Stockholm Public Health Cohort in 2002.
Sampling strategy	No sampling strategy. All eliglbe members of the population at-risk were included.
Data collection	Data on our outcome and confounders were obtained from the linked national Swedish registries, including the following registers: total population, national patient register, multigenerational register, immigration/emigration register (STATIV), and the labour market register (LISA). All register data were recorded for administrative purposes. Data on our exposure came from the Stockholm Public Health Cohort respondents in 2002. The instrument used was a 14-item instrument to capture aspects of trust in different groups, communities or institutions and voting behaviour. Those involved in data collection would have been blinded to this study and its hypotheses which were conceived after data collection.
Timing	2002-2016, inclusive.
Data exclusions	We excluded people who were diagnosed with any outcome of interest before the beginning of follow-up. We also excluded people born outside of Sweden but who later migrated to Sweden. Any participant who did not live in our study region (Stockholm County) during the date and age ranges specified in our study were also excluded.
Non-participation	OF 1,527,279 eligible participants, we excluded 3.9% of the sample (N=60,151) due to missing data on exposure or covariates.
Randomization	None. Control for covariates was detemrined a priori via directed acyclic graphs and during sensitivity analyses via instrumental variable approaches.

Reporting for specific materials, systems and methods

We require information from authors about some types of materials, experimental systems and methods used in many studies. Here, indicate whether each material, system or method listed is relevant to your study. If you are not sure if a list item applies to your research, read the appropriate section before selecting a response.

Materials & experimental systems

n/a	Involved in the study
☒	☐ Antibodies
☒	☐ Eukaryotic cell lines
☒	☐ Palaeontology and archaeology
☒	☐ Animals and other organisms
☒	☐ Clinical data
☒	☐ Dual use research of concern
☒	☐ Plants

Methods

n/a	Involved in the study
☒	☐ ChIP-seq
☒	☐ Flow cytometry
☒	☐ MRI-based neuroimaging

Plants

Seed stocks

Report on the source of all seed stocks or other plant material used. If applicable, state the seed stock centre and catalogue number. If plant specimens were collected from the field, describe the collection location, date and sampling procedures.

Novel plant genotypes

Describe the methods by which all novel plant genotypes were produced. This includes those generated by transgenic approaches, gene editing, chemical/radiation-based mutagenesis and hybridization. For transgenic lines, describe the transformation method, the number of independent lines analyzed and the generation upon which experiments were performed. For gene-edited lines, describe the editor used, the endogenous sequence targeted for editing, the targeting guide RNA sequence (if applicable) and how the editor was applied.

Authentication

Describe any authentication procedures for each seed stock used or novel genotype generated. Describe any experiments used to assess the effect of a mutation and, where applicable, how potential secondary effects (e.g. second site T-DNA insertions, mosiacism, off-target gene editing) were examined.
